# Mapping programmable mRNA research relevant to cancer-associated immune escape: a dual-database bibliometric and topic modeling analysis

**DOI:** 10.3389/fonc.2026.1957469

**Published:** 2026-10-07

**Authors:** Xiao-Jie Zhou, Jun-Chi Zhu, Jian Dong, Shi-Ying Yan, Si-Yin Zhang, Jun-Ting Dong, Bai-Qi Zhang, Hong-Ru Che, Chen-Hao Zhang, Rui Zhang, Chi Zhang, De-Zhu Liu, Cheng-Gang Liu

**Affiliations:** 1 First Clinical Medical College, Heilongjiang University of Chinese Medicine, Harbin, Heilongjiang, China; 2 Second Clinical Medical College, Heilongjiang University of Chinese Medicine, Harbin, Heilongjiang, China; 3 Department of Basic Medical Science, Heilongjiang University of Chinese Medicine, Harbin, Heilongjiang, China; 4 Graduate School, Heilongjiang University of Chinese Medicine, Harbin, Heilongjiang, China

**Keywords:** BERTopic, bibliometric analysis, cancer vaccine, lipid nanoparticle, neoantigen, programmable mRNA, topic modeling, tumor immune escape

## Abstract

**Background:**

Programmable messenger RNA (mRNA) platforms can encode tumor antigens or immunomodulators, enabling coordinated antigen delivery and immune modulation. Yet the literature spans cancer vaccination, delivery engineering, immune biology, and adjacent infectious-disease research, making its structure and evidentiary boundaries difficult to discern. We mapped this cross-domain corpus to clarify its development, organization, and relevance to cancer-associated immune escape.

**Methods:**

We searched the Web of Science Core Collection (WoSCC) and Scopus on July 29, 2026, for English-language Articles and Reviews published since 2006. The strategy combined programmable or therapeutic mRNA, cancer, and immune-escape-related concepts. R-based analyses summarized the number of publications (NP), total citations (TC), and collaboration patterns; BERTopic modeled titles and abstracts after entity-aware preprocessing. Independent two-author review supported the five topic labels and characterized the outliers, and robustness was assessed across alternative clustering configurations.

**Results:**

The dataset comprised 756 publications (NP = 756). Annual output increased more than fourfold in 2021 and reached NP = 182 in 2025. China (NP = 291; 38.49%) and the United States (NP = 146; 19.31%) accounted for nearly 58% of corresponding-author publications. Sichuan University and Frontiers in Immunology ranked first in institutional and journal output, respectively. Nature had the highest retained source-record citation total (TC = 2,326). BERTopic assigned 745 publications to five topics, while 11 of 756 records remained outliers (1.46%). The topics represented mRNA delivery and immunomodulatory cancer-vaccine research (52.1%), SARS-CoV-2 vaccination in oncology and immunotherapy settings (23.6%), therapeutic mRNA cancer-vaccine platforms (13.3%), tumor-antigen discovery and immune-subtype stratification (8.6%), and mRNA-vaccine immunogenicity during autoimmune immunosuppression (2.4%).

**Conclusions:**

Publication activity in the retrieved cross-domain corpus expanded rapidly after 2020. The corpus included both direct cancer-mRNA research and adjacent platform or immune-response literature. By distinguishing these components and identifying thematic connections among delivery, antigen selection, immune context, and collaboration, the analysis provides an evidence-navigation framework for prioritizing systematic reviews, mechanistic studies, and clinical evaluation. The map does not itself establish therapeutic efficacy.

## 1 Introduction

Cancer immunotherapy has changed the treatment of many malignancies, but its benefit is often limited by tumor immune escape (1). Tumors can evade immune recognition or destruction through overlapping mechanisms, including increased PD-L1 expression, PD-1 and CTLA-4 signaling, impaired major histocompatibility complex (MHC) class I antigen presentation, secretion of immunosuppressive mediators, and recruitment of regulatory T cells and myeloid-derived suppressor cells (1–5). Immune checkpoint inhibitors (ICIs) counter some of these mechanisms, but response rates vary substantially by tumor type, treatment setting, and agent, and durable benefit is limited to a subset of patients (2, 4–6). Chimeric antigen receptor (CAR)-T-cell therapy has produced substantial responses in selected hematologic malignancies but remains less effective in most solid tumors because of antigen heterogeneity, an immunosuppressive tumor microenvironment, and restricted T-cell infiltration (7, 8). These limitations support continued investigation of approaches that broaden tumor recognition and modulate local immunity.

In this study, programmable mRNA refers to engineered transcripts designed to express one or more tumor antigens, neoantigens, or immunomodulatory proteins. An mRNA construct typically contains a coding sequence flanked by untranslated regions, a 5′ cap, and a poly(A) tail; modified nucleosides can improve translation and reduce unwanted innate sensing (9–13). Lipid nanoparticles protect mRNA from extracellular degradation and facilitate cellular uptake, although tissue distribution and endosomal escape remain formulation-dependent (14–19). After cytosolic release, translated antigens can enter MHC class I and II presentation pathways and support CD8+ and CD4+ T-cell priming. Personalized cancer vaccines target selected tumor neoantigens, whereas programmable mRNA constructs can encode cytokines, costimulatory molecules, or checkpoint-modulating proteins (20–26).

These platform components interact with tumor immune escape at several levels. Vaccine-induced T cells may be limited by antigen heterogeneity, impaired MHC class I presentation, PD-1/PD-L1 or CTLA-4 signaling, T-cell exclusion or exhaustion, and suppressive regulatory T cells or myeloid-derived suppressor cells (1–5, 27–31). Programmable mRNA may address selected barriers by broadening antigenic targets, restoring or enhancing immune stimulation, or encoding locally active immunomodulators. However, the biological effect depends on payload, target cell, delivery site, expression kinetics, and tumor context; an mRNA platform does not automatically reverse immune escape.

Bibliometric analysis examines publication metadata, including authorship, affiliations, sources, citations, and keywords, to characterize a research corpus. R is an open-source statistical computing environment, and bibliometrix is an R package for bibliographic analysis and science mapping (32, 33). Deduplication removes overlapping database records. BERTopic groups contextually similar title–abstract texts into document clusters, termed topics, while low-density records that remain unassigned are termed outliers (34).

The literature linking programmable mRNA, cancer, and immune-escape-related concepts spans oncology, delivery science, vaccine research, and immune-response studies. Existing cancer RNA-vaccine mapping has not fully resolved how these overlapping domains are organized across databases or how directly each component relates to tumor immune escape (34, 35). A cross-domain evidence map is therefore needed to distinguish cancer-focused research from adjacent platform and immunology literature.

To address this gap, we integrated WoSCC and Scopus and combined bibliometric mapping with contextual topic modeling. We characterized publication growth, collaboration, source distribution, and thematic evolution from 2006 to 2026. The analysis was designed to organize the cross-domain evidence base and identify priorities for focused review and experimentation, not to imply that every retrieved record directly evaluated therapeutic reversal of tumor immune escape.

## 2 Materials and methods

### 2.1 Data sources and search strategy

Records were retrieved from the Web of Science Core Collection (WoSCC) and Scopus on July 29, 2026. The search combined three Boolean concept blocks covering programmable or therapeutic mRNA, cancer or neoplasms, and immune-escape-related mechanisms or responses. Synonyms within each block were combined with OR, and the three blocks were combined with AND. We searched the Topic Search field in WoSCC and TITLE-ABS-KEY in Scopus for records published from January 1, 2006, through the search date. The 2006 start date defined a 20-year observation window focused on contemporary programmable mRNA development and modern cancer immunotherapy. The immune block intentionally included checkpoint terms together with T-cell activation, T-cell response, and antigen presentation because immune escape can arise from checkpoint signaling, impaired immune recognition, or ineffective effector responses. This broad operationalization favored sensitivity over specificity. Complete database-specific queries are provided in **Supplementary Table 1**. The searches identified 458 WoSCC records and 761 Scopus records. Eligibility was restricted to English-language Articles and Reviews. In WoSCC, 26 records were excluded because they did not meet the prespecified language or document-type criteria. In Scopus, 50 records outside the Article or Review categories and one non-English record were excluded. This left 432 WoSCC records and 710 Scopus records. Two researchers independently checked selection and data verification, and a third resolved any disagreement.

Records from the two databases were harmonized and deduplicated using a standardized automated workflow with manual review. Deduplication was defined as identifying records that represented the same publication across the two sources. Scopus fields were first mapped to a WoSCC-compatible structure. Normalized Digital Object Identifiers (DOIs) served as the primary matching key, and the WoSCC record was retained when both databases indexed the same DOI. Normalized titles flagged records without a shared DOI for manual review; these candidates were retained unless duplication was confirmed. The workflow removed 386 shared-DOI records. The final dataset contained 756 publications (NP = 756), including 559 Articles and 197 Reviews, and was exported in WoSCC plain-text format.

The Preferred Reporting Items for Systematic Reviews and Meta-Analyses (PRISMA) flow diagram provides a structured account of record identification, eligibility assessment, deduplication, exclusion, and final inclusion (36). **Figure 1** presents these steps as a PRISMA-informed bibliometric workflow. Reporting completeness was additionally assessed with the BIBLIO checklist for biomedical bibliometric reviews, provided as **Supplementary Table 2**.

**Figure 1:**
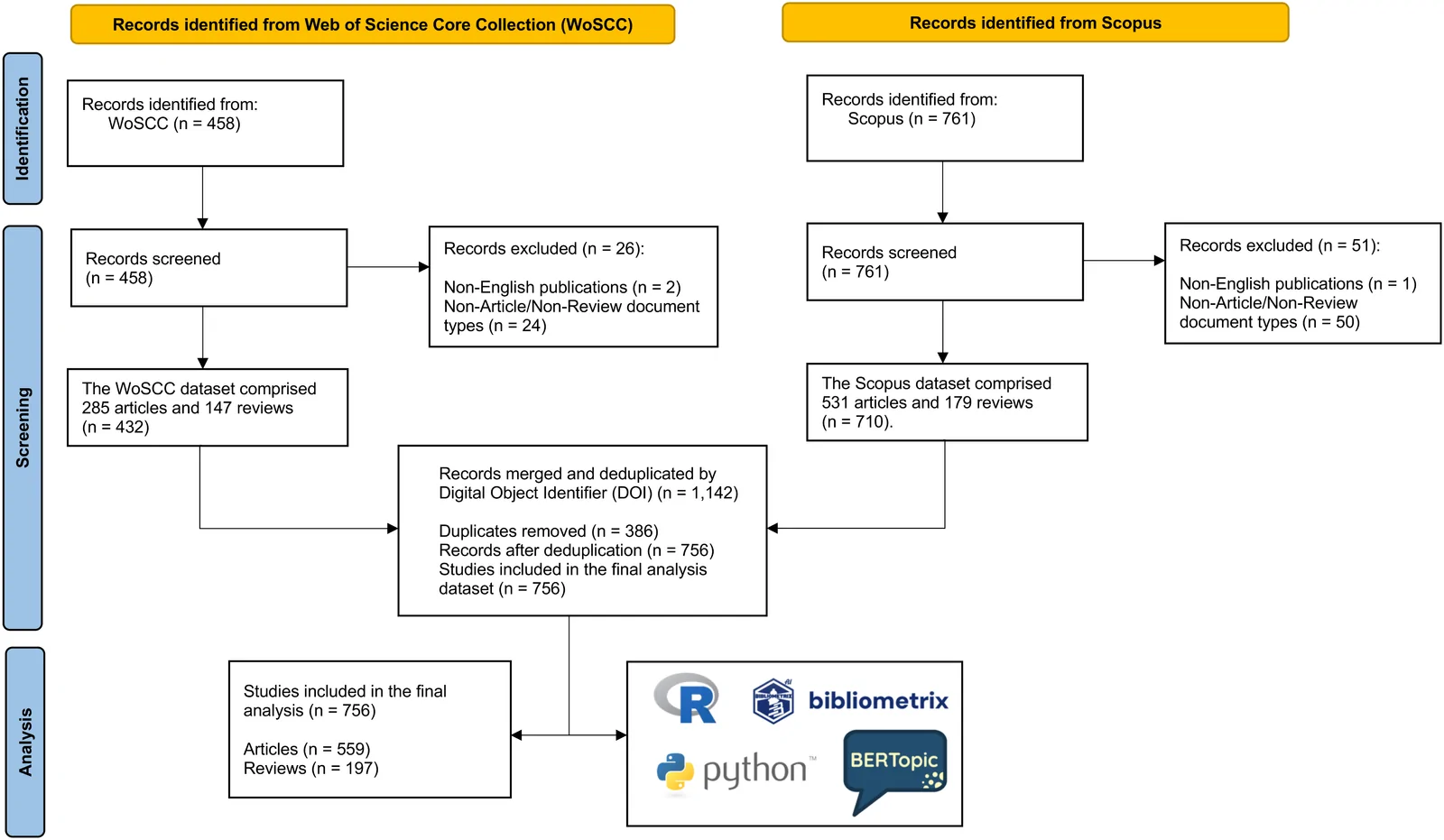
Literature retrieval, screening, deduplication, and inclusion workflow. The searches identified 458 WoSCC records and 761 Scopus records. After language and document-type filtering, 432 WoSCC and 710 Scopus records remained. The 1,142 retained records were merged, 386 cross-database duplicates were removed, and the final analysis file contained 756 publications, including 559 Articles and 197 Reviews.

### 2.2 Data preprocessing and bibliometric analysis

The merged dataset was parsed and standardized in R (version 4.5.2), an open-source statistical computing environment. A thesaurus consolidated selected keyword variants. For example, “mRNA vaccines” and “mRNA vaccination” were grouped under “mRNA vaccine”, whereas “SARS-CoV-2”, “COVID-19”, and “coronavirus disease 2019” were grouped under “COVID-19” for the specified keyword analyses. Generic database terms without substantive thematic meaning, such as “human” and “article”, were removed. Canonical capitalization was retained for biomedical abbreviations and named entities. After cleaning, 22,627 keyword entries were available. Author keywords and database-supplied index terms were analyzed together after harmonization. Institutional affiliations were standardized by consolidating selected secondary units under their parent organizations, and common variants of author and institutional names were normalized.

Bibliometric analyses were performed with bibliometrix (version 5.4.1), ggplot2, circlize, and related R packages (32, 37). Publication output was expressed as the number of publications (NP), and citation impact as total citations (TC). Annual NP and TC were displayed together, and a second-order polynomial provided a descriptive summary of publication growth. Corresponding-author country output was divided into single-country publications (SCPs) and multiple-country publications (MCPs). For the geographic participation map, each publication contributed one count to every country represented in its author affiliations; Hong Kong and Taiwan affiliations were harmonized with China for this country-level display. International collaboration was summarized by identifying the unique countries represented in each publication, counting each unordered country pair at most once per publication, and aggregating the resulting pair counts. The 25 countries with the highest aggregate pair counts were displayed in the chord diagram. Institutional contributions used full counting after affiliation-name standardization and within-record deduplication, so each standardized institution received no more than one publication credit per record. For institutional collaboration, each unordered institution pair was counted at most once per publication. Redundant umbrella-system entries were removed when the corresponding member university appeared in the same record, and the 25 institutions with the highest aggregate pair counts were displayed in the chord diagram. Indexed surname–initial strings were used for author output and co-authorship analyses because complete identifier-based disambiguation was unavailable. Local H-, G-, I10-, and M-indices were calculated only within the retrieved dataset; the M-index used the first publication year represented for each indexed name string. Journal analyses included NP, retained source-record TC, 2025 Journal Impact Factors (JIFs), and 2025 Journal Citation Reports (JCR) quartiles. For each retained record, the citation-count source was identified as either WoSCC or Scopus. Citation-based comparisons were descriptive because WoSCC and Scopus use different citation-counting systems. Keyword analyses comprised a factorial map, trend-topic plot, annual frequency heatmap, and co-occurrence network.

### 2.3 Statistical analysis

The publication record was the independent unit for statistical comparison. Because author counts and cited-reference counts were right-skewed, Articles and Reviews were compared using two-sided Wilcoxon rank-sum tests. Records lacking the corresponding indexed value were excluded from each comparison. Continuous variables are reported as medians with interquartile ranges (IQRs). Rank-biserial correlation (*r*_rb_) was reported as the effect size; positive values indicate higher values for Articles than Reviews. The two comparisons were prespecified, and no multiplicity adjustment was applied. Statistical significance was defined as a two-sided P value < 0.05. Figure annotations denote *P < 0.05, **P < 0.01, ***P < 0.001, and ****P < 0.0001. The numbers of authors and cited references were displayed using violin plots with embedded box plots. No inferential test was applied to the polynomial trend or topic prevalence. Analyses were performed in R (version 4.5.2).

### 2.4 BERTopic-based topic modeling

Titles and abstracts were concatenated for topic modeling; the title alone was used for the single record without an abstract. Text was lowercased and normalized while preserving key biomedical entities, including PD-1, PD-L1, CTLA-4, IL-2, CD8, MHC, and MHC class I. Hyphenated and spaced variants were harmonized, and no blanket token-length filter was applied. Stop words were retained during semantic embedding but excluded from topic-term weighting. All 756 records were processed identically.

BERTopic (version 0.17.4) identified contextual themes from title–abstract text (38). The all-MiniLM-L6-v2 sentence-transformer generated document embeddings (39), UMAP reduced their dimensionality (40), and HDBSCAN identified dense document clusters and low-density outliers (41). Topic descriptors were calculated from one- and two-word terms with BERTopic’s standard class-based term frequency–inverse document frequency (c-TF-IDF) transformation. Documented lexical variants were harmonized before applying the same transformation. The inverse-frequency component used each term’s total frequency across the topic-aggregated documents. The primary analysis used a five-dimensional UMAP representation and a minimum HDBSCAN cluster size of 12. Complete settings are reported in the **Supplementary Methods**.

The primary model produced four non-outlier topics and 11 outliers, with 487 publications concentrated in the largest topic. A prespecified secondary clustering step was applied to this dominant cluster to improve thematic resolution. Within this step, 62 low-density records were reassigned to a secondary topic by model transformation and a highest-membership-probability fallback. The 11 primary-model outliers remained unassigned. Combining the secondary subtopics with the remaining primary topics yielded five topics among 745 assigned publications. Topic labels were based on weighted terms and representative titles. Primary-model outliers were excluded from topic-specific prevalence and temporal analyses. Full specifications are provided in the **Supplementary Methods**.

Topic prevalence was calculated using the 745 assigned publications as the denominator. Intertopic relationships were assessed by multidimensional scaling of cosine distances between weighted topic–term profiles. The five highest-weight harmonized terms were identified for each topic, and annual values were normalized within topics. Cosine similarity was also calculated among frequently occurring, harmonized document-supplied keywords.

Co-occurrence among harmonized document-supplied keywords was analyzed using the 745 assigned publications. Keyword fields were linked to topic assignments by normalized DOI or, when a DOI was unavailable, by a unique title–year–abstract key; one-to-one matching was required. Nodes were selected using document frequency and topic enrichment, and edges represent document-level co-occurrence. A maximum-spanning backbone plus the strongest remaining connections was retained for readability. Node size represents frequency, color indicates the topic of greatest within-topic prevalence, and position was fixed for label readability rather than semantic distance. Full selection thresholds are provided in the **Supplementary Methods**.

Topic modeling was performed in Python 3.14.2 with BERTopic 0.17.4, sentence-transformers 5.6.1, umap-learn 0.5.12, hdbscan 0.8.44, and scikit-learn 1.9.0. A fixed seed was used where supported to ensure reproducibility.

Two authors independently reviewed representative titles and abstracts from each of the five final topics and all 11 outliers. Consensus review supported the topic labels and the distinction between cancer-focused and adjacent literature. The review procedure is described in the **Supplementary Methods**, and the 11 outliers are listed in **Supplementary Table 3**.

Topic stability was examined across prespecified model initializations and minimum cluster sizes. Assignment agreement was evaluated using the adjusted Rand index (ARI) and normalized mutual information (NMI). Complete configurations and results are reported in **Supplementary Methods** and **Supplementary Table 4**.

To assess the effect of revised preprocessing, we compared the selected entity-aware workflow with the originally submitted rule that removed all one- and two-character tokens. ARI and NMI quantified agreement between the two document partitions. This comparison reflects the combined effect of entity normalization and removal of the blanket length filter.

## 3 Results

### 3.1 Annual publication trends and characteristics of document types

The dataset comprised 756 publications (NP = 756) published between January 1, 2006, and July 29, 2026 (**Figure 2C**). Annual output remained low and variable from 2006 through 2020 (NP ≤ 10 per year). It increased from NP = 10 in 2020 to NP = 47 in 2021 and reached NP = 182 in 2025. The 2026 value (NP = 148) represents a partial year and is not directly comparable with complete annual counts. The descriptive polynomial was fitted to all calendar years from 2006 through 2026, including the four years with NP = 0. Here, x represents the calendar year itself (e.g., x = 2006), with no baseline subtraction or centering. The fitted curve was y = 0.9224494041x² − 3711.808205x + 3,733,936.993 (R² = 0.909). The fit was not used for forecasting or causal inference. Annual citation totals peaked for papers published in 2023 (TC = 4,423); lower TC values for 2024–2026 reflect shorter citation windows.

**Figure 2:**
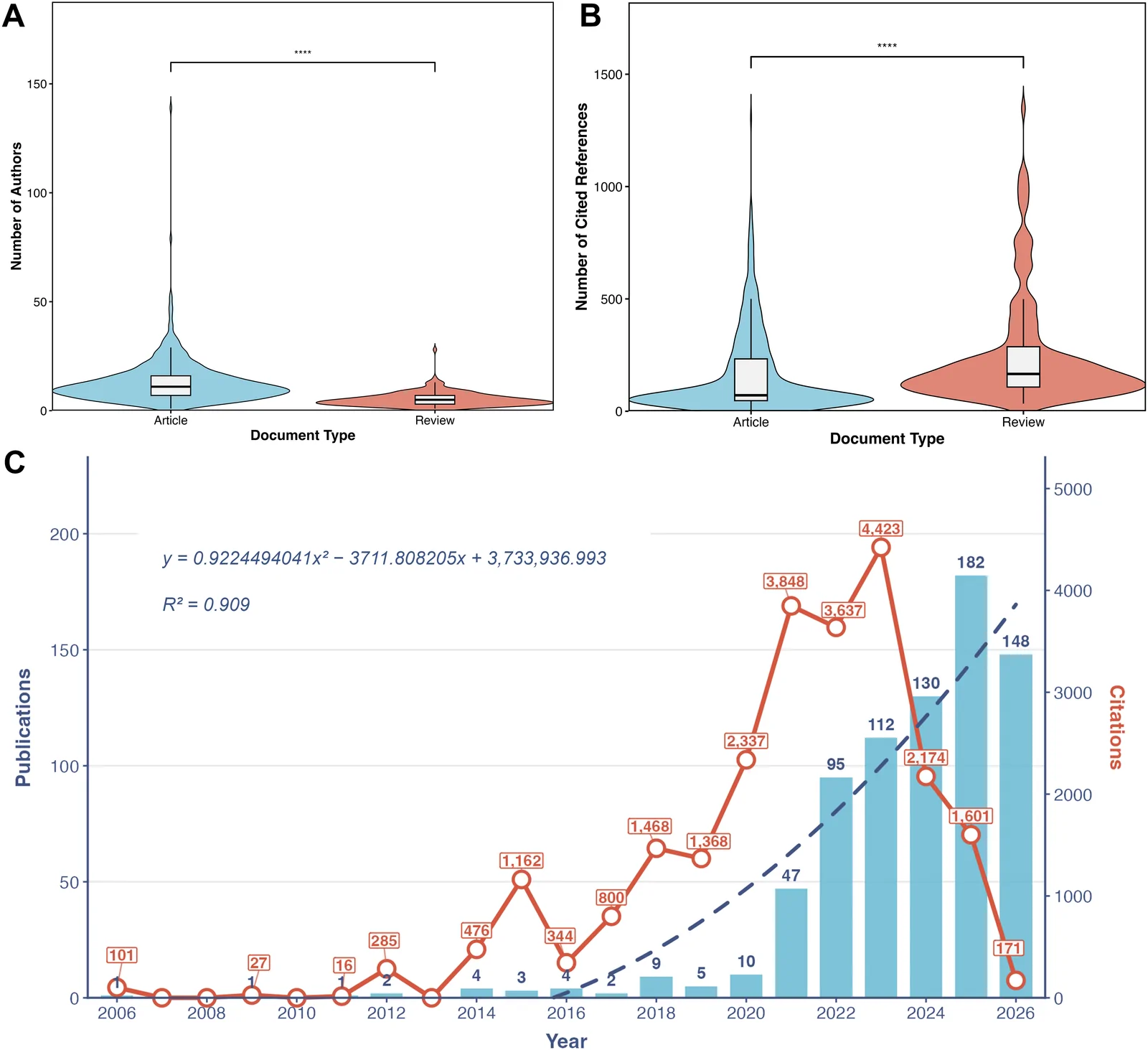
Characteristics of document types and annual publication trends. **(A)** Distribution of the number of authors per Article and Review. **(B)** Distribution of cited-reference counts per Article and Review. Violin plots show the distributions, with embedded box plots indicating medians and interquartile ranges. Comparisons used two-sided Wilcoxon rank-sum tests. The author-count comparison included 558 Articles and 197 Reviews because one Article lacked indexed author data; the cited-reference comparison included 555 Articles and 195 Reviews after six records with missing indexed values were excluded. Rank-biserial correlations were 0.692 for author count and −0.387 for cited-reference count (Article versus Review); ****P < 0.0001. **(C)** Annual NP (bars) and TC attached to publications from each year at database export (line), covering 2006 through July 29, 2026. Recent publication years have shorter citation windows, and 2026 is a partial year. The dashed curve shows the descriptive second-order polynomial fitted to all calendar years from 2006 through 2026, including years with NP = 0. Here, x represents the calendar year itself, with no baseline subtraction or centering. The fitted equation is y = 0.9224494041x² − 3711.808205x + 3,733,936.993 (R² = 0.909).

Article and Review characteristics are shown in **Figures 2A, B**. Articles had more authors than Reviews (median [IQR], 11 [7–16] vs. 5 [3–7]; P < 0.0001; *r*_rb_ = 0.692), whereas Articles cited fewer references (71 [47–233] vs. 166 [107.5–287]; P < 0.0001; *r*_rb_ = −0.387). The distributions were right-skewed, with several highly collaborative Articles and Reviews containing unusually large reference lists.

### 3.2 Country contributions and international collaboration networks

Corresponding-author countries are summarized in **Table 1** and **Figure 3A**. China had the largest output (NP = 291; 38.49%), followed by the United States (NP = 146; 19.31%). Together, they accounted for 57.80% of the dataset. Italy ranked third (NP = 46; 6.08%), followed by South Korea (NP = 25; 3.31%), Germany (NP = 24; 3.17%), Belgium (NP = 22; 2.91%), and Japan (NP = 21; 2.78%).

**Table 1:** Top 15 Corresponding-author countries by publication output.

Rank	Country	NP	NP %	SCP	MCP	MCP %
1	China	291	38.49	263	28	9.62
2	United States	146	19.31	124	22	15.07
3	Italy	46	6.08	38	8	17.39
4	South Korea	25	3.31	22	3	12.00
5	Germany	24	3.17	20	4	16.67
6	Belgium	22	2.91	21	1	4.55
7	Japan	21	2.78	20	1	4.76
8	Australia	14	1.85	10	4	28.57
9	France	14	1.85	12	2	14.29
10	India	12	1.59	11	1	8.33
11	Iran	10	1.32	10	0	0.00
12	Spain	10	1.32	10	0	0.00
13	United Kingdom	10	1.32	8	2	20.00
14	Netherlands	8	1.06	8	0	0.00
15	Canada	7	0.93	6	1	14.29

NP, number of publications; SCP, single-country publications; MCP, multiple-country publications. Country assignment was based on the corresponding author’s affiliation.

**Figure 3:**
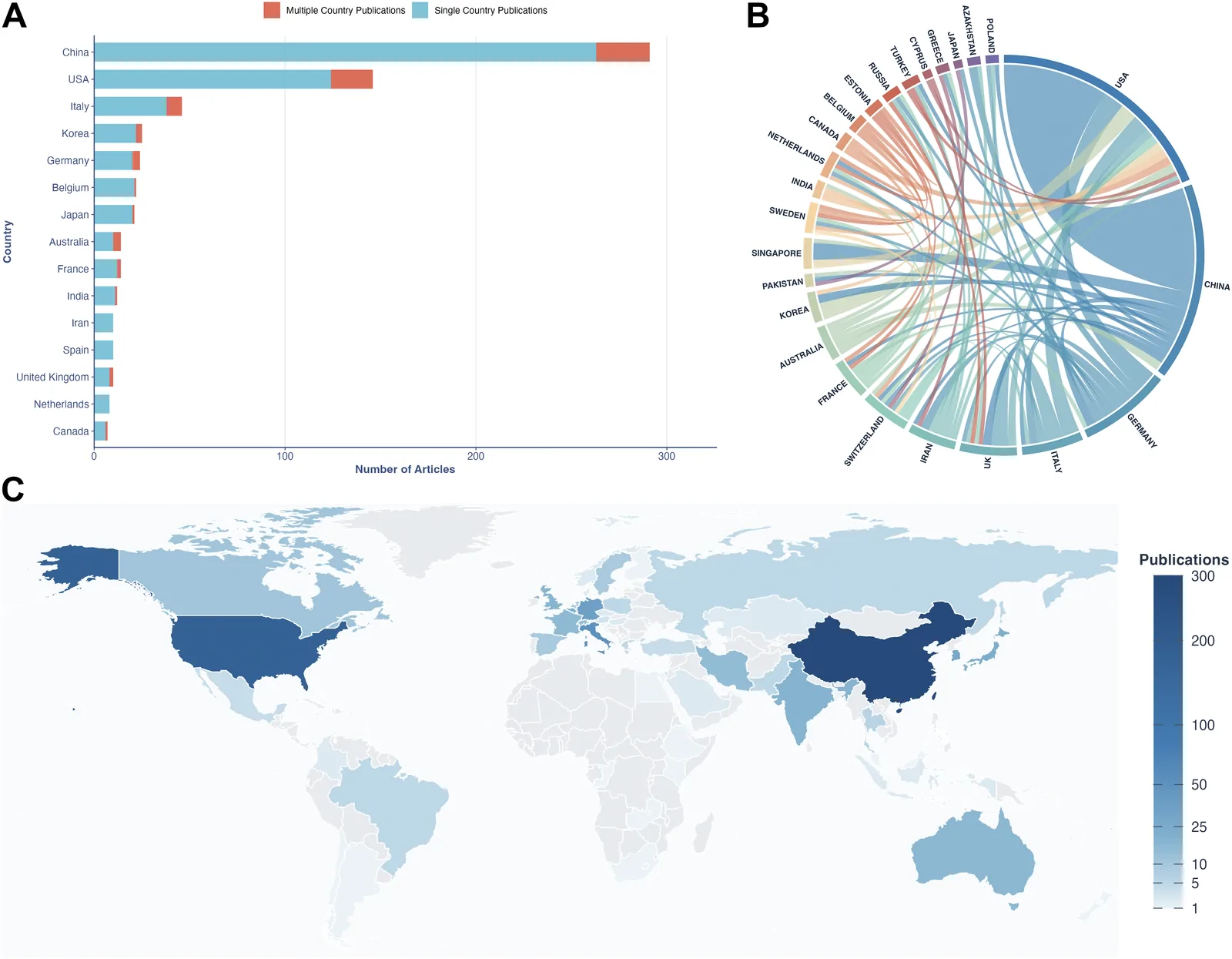
Country output, collaboration, and geographic participation. **(A)** Publications from the 15 leading corresponding-author countries, divided into single-country publications and multiple-country publications. **(B)** Chord diagram for the 25 countries with the highest aggregate pair counts. Each publication contributes at most one count to an unordered country pair, and each ribbon represents that pair after aggregation across the corpus; ribbon width indicates the number of internationally coauthored publications. **(C)** Geographic heat map based on all author affiliations. Each publication contributes one count to every represented country, so these full-participation counts differ from the corresponding-author counts in panel **(A)**. Darker shading indicates greater participation. Hong Kong and Taiwan affiliations were harmonized with China for panels **(B, C)**.

China had 263 SCPs and 28 MCPs, corresponding to an MCP proportion of 9.62%. Australia had the highest MCP proportion among the 15 countries shown (28.57%), followed by the United Kingdom (20.00%), Italy (17.39%), Germany (16.67%), and the United States (15.07%). These proportions describe the corresponding-author classification used in this analysis and should not be interpreted as direct measures of national research quality.

The Top-25 chord diagram showed that China and the United States occupied the largest sectors and were linked to multiple international partners; additional participation was visible across European and Asian countries (**Figure 3B**). The full-participation geographic map covered 54 countries and showed the greatest publication activity in China and the United States, with broader participation across Europe, Asia, Oceania, and the Americas (**Figure 3C**). Because the map assigns one count to every country represented in a publication’s author affiliations, its full-participation pattern differs from the corresponding-author counts in **Figure 3A** and **Table 1**.

### 3.3 Institutional contributions and collaboration networks

The full-counting institutional analysis is presented in **Table 2** and **Figure 4A**. Each standardized institution received no more than one publication credit per record. Sichuan University had the highest output (NP = 33), followed by the Chinese Academy of Sciences (NP = 32), Sun Yat-sen University and Zhejiang University (NP = 20 each), and Harvard University (NP = 18). Among the 10 leading institutions, Peking University had the highest mean citation count per publication (72.5), followed by Vrije Universiteit Brussel (64.1), Harvard University (63.1), and Sun Yat-sen University (51.7). The ranking mixes standalone universities with umbrella or system-level organizations, so counts are not directly comparable as measures of institution-level productivity. Citation means are also descriptive because their distributions depend on publication year and document type.

**Table 2:** Top 10 Institutions by full-counting output.

Rank	Institution	NP	TC	Avg. citations
1	Sichuan University	33	853	25.8
2	Chinese Academy of Sciences	32	445	13.9
3	Sun Yat-sen University	20	1,034	51.7
4	Zhejiang University	20	816	40.8
5	Harvard University	18	1,135	63.1
6	University of California	18	317	17.6
7	Chinese Academy of Medical Sciences - Peking Union Medical College	18	205	11.4
8	Vrije Universiteit Brussel	15	962	64.1
9	Shanghai Jiao Tong University	13	300	23.1
10	Peking University	11	797	72.5

NP, full-counting institutional output; TC, total citations; Avg. Citations, mean citations per publication (TC/NP). Affiliation variants were standardized before analysis, and each standardized institution received no more than one publication credit per record. Counts should be interpreted together with the limitations of institutional aggregation described in the manuscript.

**Figure 4:**
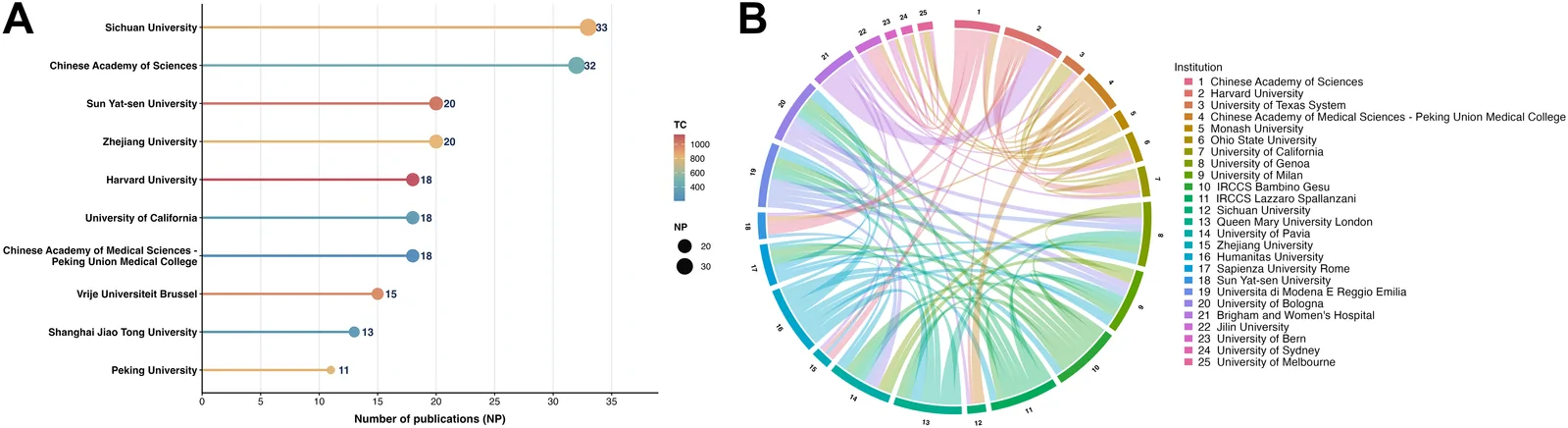
Institutional output and collaboration. **(A)** Full-counting output for the 10 leading standardized institutional affiliations; each institution received no more than one publication credit per record. Marker size represents NP, and color represents TC. **(B)** Chord diagram of institutional collaboration among the 25 institutions with the highest aggregate pair counts. Each unordered institution pair contributed at most once per publication; ribbon width represents the aggregated number of coauthored publications.

**Figure 4B** summarizes collaboration among the 25 institutions with the highest aggregate pair counts. Frequently represented Chinese institutions included the Chinese Academy of Sciences, Sichuan University, Zhejiang University, Sun Yat-sen University, and Jilin University. Harvard University, the University of California, the University of Texas System, and Ohio State University were prominent among the selected US institutions. Italian institutions, including the University of Milan, the University of Pavia, Sapienza University of Rome, and Humanitas University, formed another visible group. The diagram is descriptive and does not provide a formal test of network centrality.

### 3.4 Journal distribution and citation impact

Eleven journals published at least 10 records in the dataset (**Table 3**; **Figure 5A**). Frontiers in Immunology published the most (NP = 53; 2025 JIF, 5.9; Q1), followed by Vaccines (NP = 40; JIF, 3.4; Q2). Journal of Controlled Release published NP = 21, and Molecular Therapy published NP = 15. Molecular Cancer had the highest 2025 JIF among these 11 journals (33.9) and published NP = 12.

**Table 3:** Eleven journals with the highest publication output.

Rank	Journal	NP	JIF (2025)	JCR quartile (2025)
1	Front. Immunol.	53	5.9	Q1
2	Vaccines	40	3.4	Q2
3	J. Control. Release	21	11.5	Q1
4	Mol. Ther.	15	11.4	Q1
5	Cancers	12	4.4	Q2
6	Front. Oncol.	12	4.2	Q1
7	Mol. Cancer	12	33.9	Q1
8	ACS Nano	11	16.0	Q1
9	Proc. Natl. Acad. Sci. USA	11	9.1	Q1
10	Int. J. Mol. Sci.	10	5.6	Q1
11	Nat. Commun.	10	15.7	Q1

NP, number of publications; JIF, 2025 Journal Impact Factor; JCR, 2025 Journal Citation Reports. Q1-Q4 indicate the reported JCR quartile. Journal quartiles may vary by subject category.

**Figure 5:**
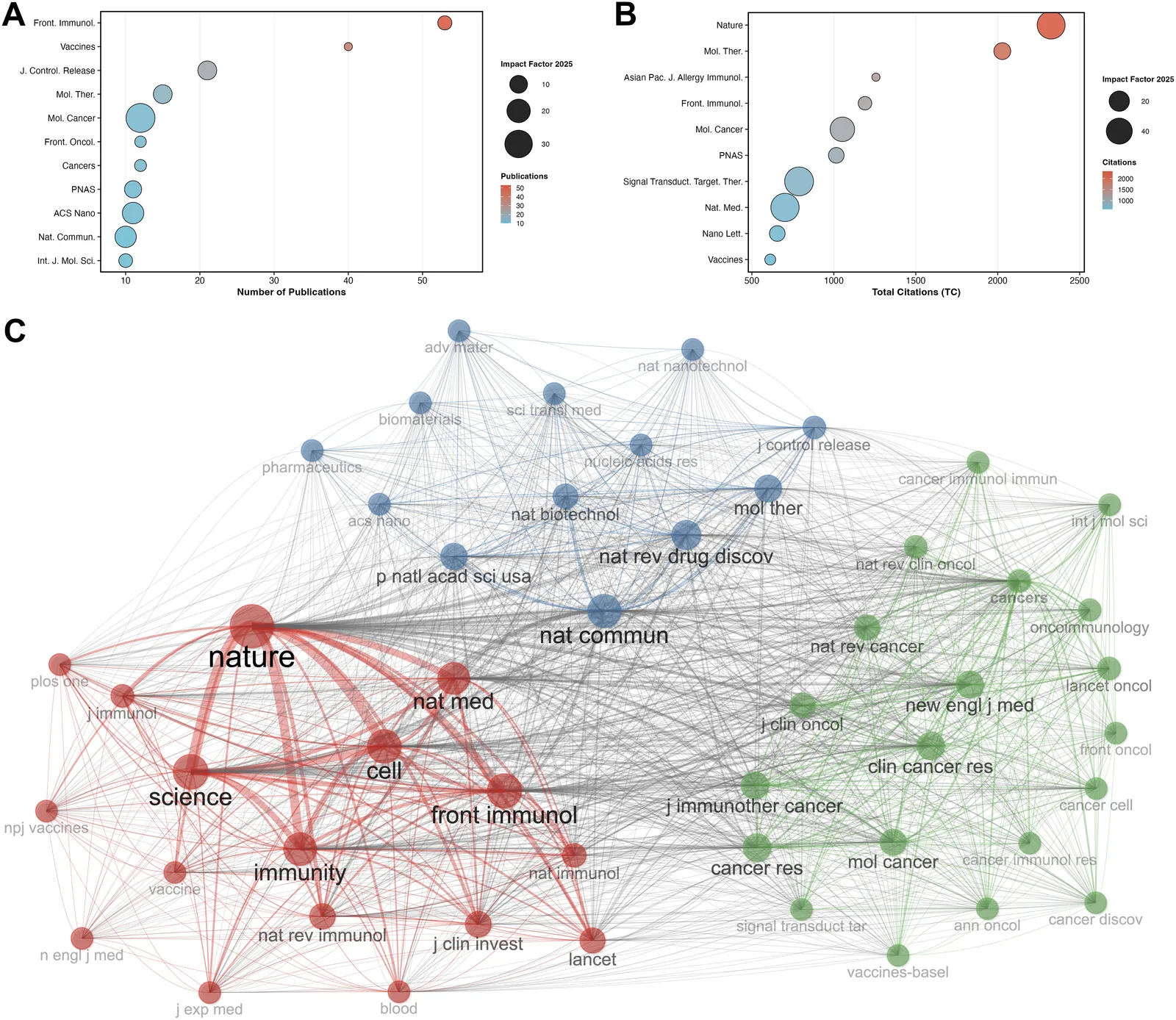
Journal distribution, retained citation totals, and co-citation structure. **(A)** Publication output of journals with at least 10 records; marker size represents the 2025 Journal Impact Factor (JIF), and color represents publication count. **(B)** Journals with the highest retained source-record citation totals; marker size represents the 2025 JIF, and color represents total citations. The plotted totals combine the citation field attached to retained WoSCC and Scopus-only records and are therefore descriptive rather than single-database comparisons. One Asian Pacific Journal of Allergy and Immunology record contributes 1,257 citations. **(C)** Journal co-citation network. Nodes represent journals, node size reflects co-citation frequency, links represent co-citation relationships, and colors identify network clusters. Journal abbreviations follow the labels displayed in the image.

When journals were ranked by retained source-record citation totals (**Table 4**; **Figure 5B**), Nature ranked first (TC = 2,326; NP = 6), followed by Molecular Therapy (TC = 2,028; NP = 15). **Table 4** reports citations per publication alongside TC and the age-standardized citation rate. One Asian Pacific Journal of Allergy and Immunology record contributed TC = 1,257 and therefore disproportionately influenced both its raw total and per-publication value. For an age-standardized descriptive check, citations per year were calculated for each record as total citations/(2026 − publication year + 1) and summed by standardized journal title. The five leading journals were Nature, Molecular Therapy, Frontiers in Immunology, Signal Transduction and Targeted Therapy, and Proceedings of the National Academy of Sciences of the United States of America; Molecular Cancer ranked sixth (**Supplementary Table 5**). **Table 4** reports the NP and TC subtotal of retained WoSCC and Scopus-only records separately. Because the citation field can originate from either database after deduplication, these measures remain source-record summaries rather than strictly single-database comparisons.

**Table 4:** Top 10 journals by retained source-record citations, citations per publication, and age-standardized citation rate.

Rank	Journal	TC	NP	WoSCC n/TC	Scopus-only n/TC	Citations/year	JIF (2025)	JCR (2025)	Citations/publication
1	Nature	2326	6	5/2,108	1/218	505.75	48.5	Q1	387.67
2	Mol. Ther.	2028	15	7/800	8/1,228	298.98	11.4	Q1	135.20
3	Asian Pac. J. Allergy Immunol.	1257	1	0/0	1/1,257	179.57	2.5	Q3	1,257.00
4	Front. Immunol.	1190	53	22/290	31/900	251.10	5.9	Q1	22.45
5	Mol. Cancer	1052	12	11/698	1/354	192.58	33.9	Q1	87.67
6	Proc. Natl. Acad. Sci. USA	1015	11	7/721	4/294	200.66	9.1	Q1	92.27
7	Signal Transduct. Target. Ther.	788	6	3/631	3/157	202.42	52.7	Q1	131.33
8	Nat. Med.	702	3	1/79	2/623	117.00	50.0	Q1	234.00
9	Nano Lett.	655	3	3/655	0/0	70.40	9.1	Q1	218.33
10	Vaccines	612	40	21/414	19/198	137.91	3.4	Q2	15.30

TC, total citations; NP, number of publications; citations/publication, TC divided by NP; n/TC, number of retained records and their citation subtotal; citations/year, sum of record-level TC/(2026 − publication year + 1); JIF, 2025 Journal Impact Factor; JCR, 2025 Journal Citation Reports quartile. Duplicate records were represented by the retained WoSCC record, while Scopus-only indicates records available only from Scopus after deduplication. Because WoSCC and Scopus citation counts are not on one scale, TC, citations/publication, and citations/year are descriptive source-record summaries.

The journal co-citation network contained three broad clusters centered on immunology, drug delivery and nanotechnology, and oncology (**Figure 5C**). *Nature Communications* connected journals across the displayed clusters, while *Nature*, *Science*, and *Cell* were among the most frequently co-cited sources.

### 3.5 Indexed author-name analysis

The 11 most frequent indexed author-name strings are listed in **Table 5** and shown in **Figure 6A**. Wang J and Wang Y each had NP = 41. Wang Y had a higher local citation total (TC = 2,086) and local H-index (16) than Wang J (TC = 1,789; H-index = 14). Li Y ranked third (NP = 38; TC = 1,119). Li Y and Li H had the highest dataset-based M-indices (2.00). These results refer to indexed surname–initial strings and may combine different individuals with the same name.

**Table 5:** Eleven most frequent indexed author-name strings.

Rank	Indexed author-name string	NP	Cited papers	TC	Avg. citations	H-index	G-index	I10-index	M-index
1	WANG J	41	34	1789	43.6	14	41	20	1.56
2	WANG Y	41	35	2086	50.9	16	41	22	1.78
3	LI Y	38	32	1119	29.4	12	33	14	2.00
4	ZHANG Y	32	26	744	23.2	12	27	14	1.50
5	CHEN Y	25	24	590	23.6	9	24	9	1.50
6	WANG X	25	19	793	31.7	12	25	12	1.50
7	LI J	24	22	541	22.5	7	23	7	0.78
8	LI H	21	20	618	29.4	12	21	12	2.00
9	LIU J	20	16	574	28.7	7	20	6	1.17
10	LIU X	20	17	342	17.1	8	18	8	1.60
11	LIU Y	20	16	247	12.3	6	15	6	1.00

Important interpretation note: all author metrics are calculated for indexed surname–initial strings, not verified individual researchers. A string can combine different people with the same surname and initial. NP, output count; TC, total citations; Avg. Citations, TC/NP. H-, G-, I10-, and M-indices are local to this dataset; the M-index uses the first year represented.

**Figure 6:**
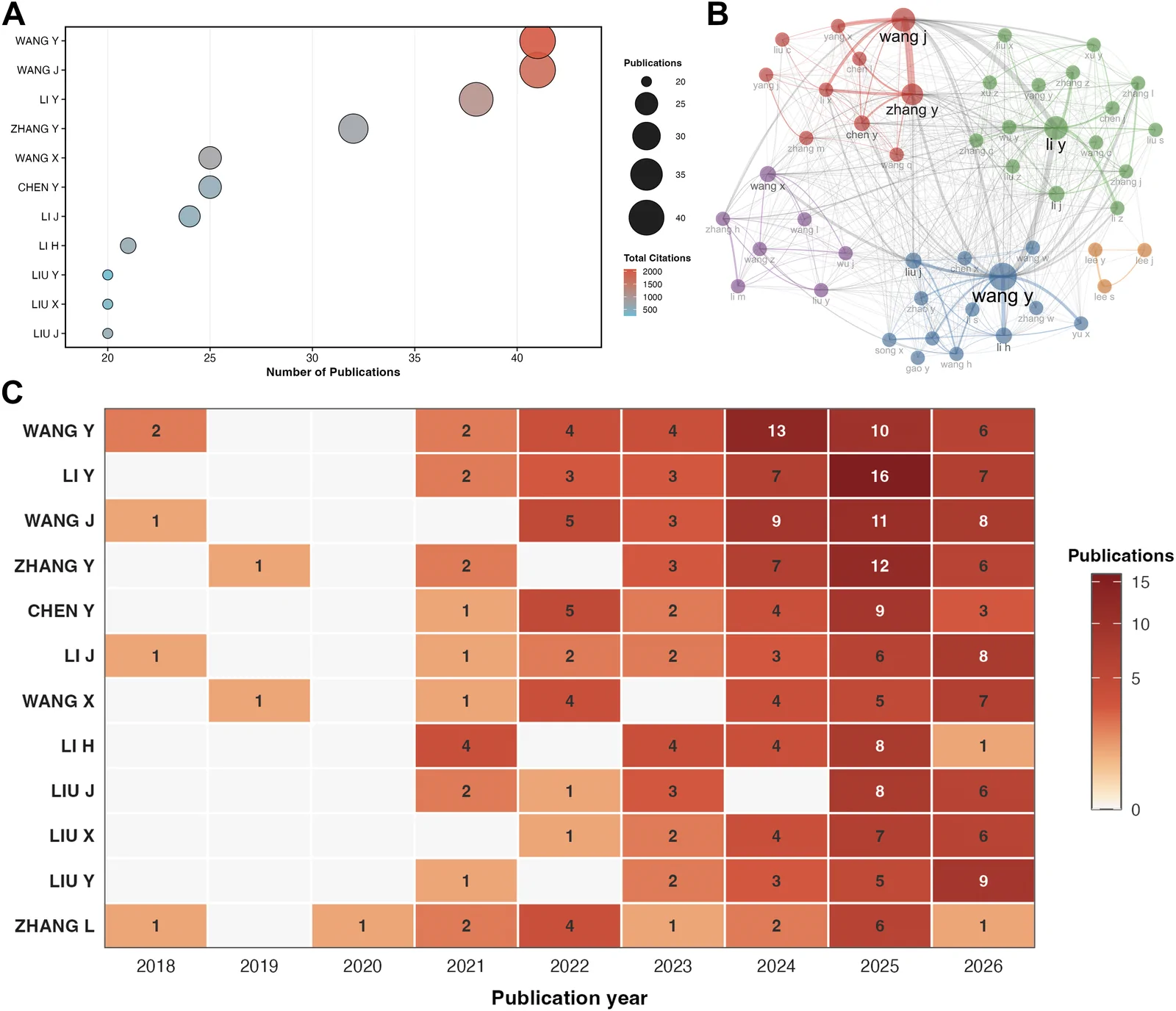
Analysis of indexed author-name strings. **(A)** Output and total citations for the 11 leading indexed surname–initial strings across the full study period; marker size represents output and color represents total citations. **(B)** Co-authorship network based on indexed name strings. Nodes represent name strings, links represent co-authorship, and colors identify network clusters. **(C)** Annual output heatmap for 12 selected high-frequency name strings from 2018 through 2026, comprising the 11 strings in panel **(A)** plus Zhang L. Because panel **(C)** begins in 2018, its row sums may be lower than the full-period totals in panel **(A)** and **Table 5**. Darker cells indicate more publications in a given year. Results are shown at the indexed name-string level and do not constitute complete author disambiguation.

The co-authorship network based on indexed name strings contained several visually distinct groups (**Figure 6B**). The largest displayed group included Wang J, Zhang Y, and Chen Y; other groups were centered on Wang Y, Li Y, and Wang X. A smaller group contained the name strings Lee J, Lee Y, and Lee S. Because identifier-based author disambiguation was incomplete, the network should be interpreted as a map of indexed co-occurrence patterns rather than a definitive map of individual researchers.

The annual heatmap showed that most high-frequency author-name strings appeared predominantly after 2020 (**Figure 6C**). **Figure 6A** and **Table 5** display the 11 leading name strings across the full study period, whereas **Figure 6C** displays 2018–2026 and includes Zhang L as a twelfth time-series row. Consequently, row sums in **Figure 6C** may be lower than the full-period totals in **Figure 6A** and **Table 5**. The highest annual output was NP = 16 for Li Y in 2025, followed by NP = 13 for Wang Y in 2024. This distribution paralleled the overall rise in annual publication output.

### 3.6 Keyword co-occurrence and thematic evolution

The factorial map (**Figure 7A**) separated several cancer-related terms, including “cancer vaccine”, “cancer immunotherapy”, and “dendritic cells”, from a group containing “COVID-19”, “SARS-CoV-2”, and “tozinameran”. Terms related to immune responses and lymphocytes occupied intermediate positions. The first two dimensions accounted for 91.7% and 7.11% of the displayed inertia, respectively.

**Figure 7:**
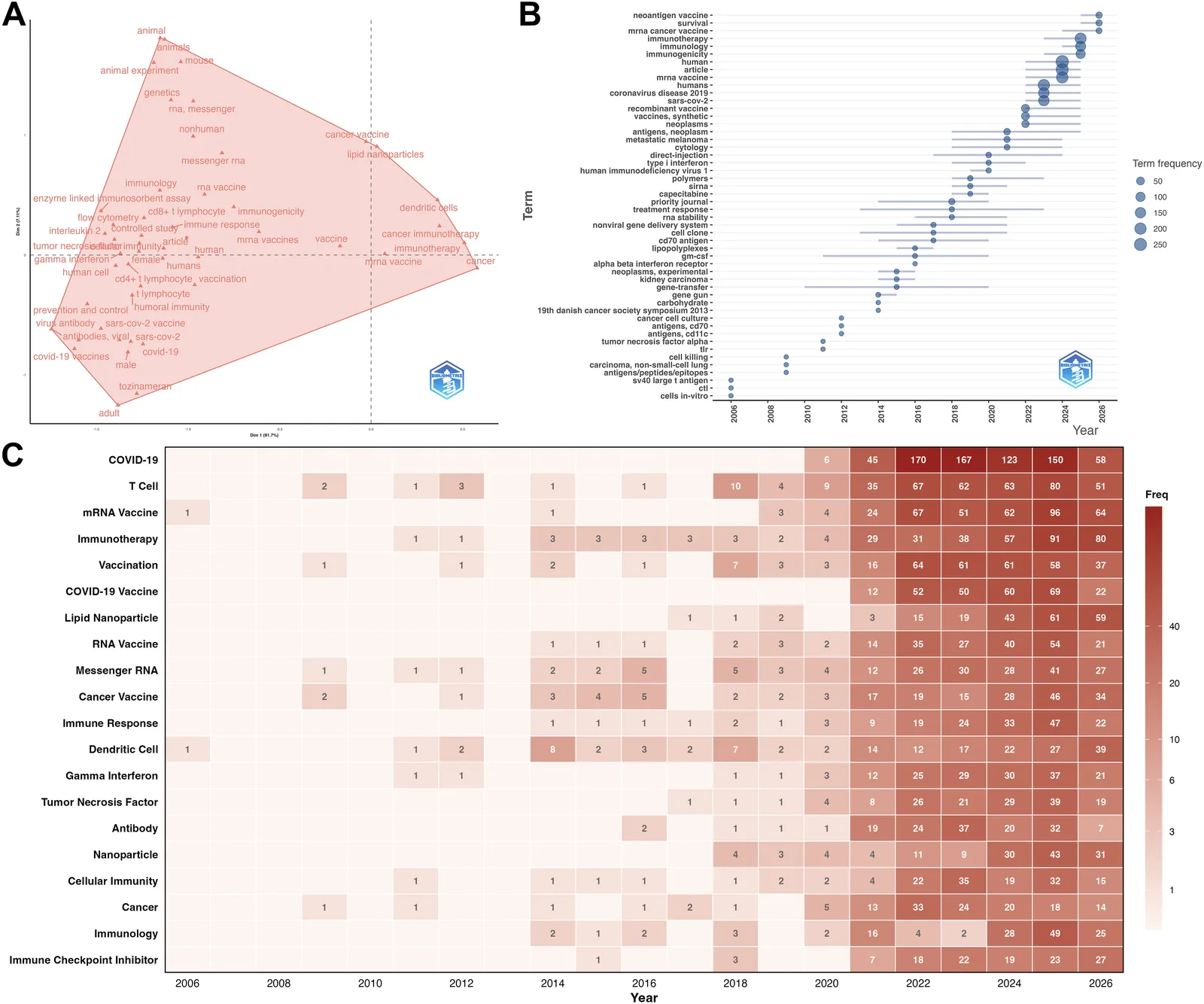
Keyword structure and temporal patterns. **(A)** Factorial map of high-frequency keywords; proximity indicates similar occurrence profiles in the analyzed corpus. **(B)** Trend-topic plot showing the time span and central year of selected terms; marker size represents term frequency. **(C)** Annual frequency heatmap for the 20 most frequent harmonized keywords. Cell labels show annual counts, and darker shading indicates higher frequency. Data for 2026 are incomplete.

The trend-topic plot summarized the periods during which selected terms were most frequent (**Figure 7B**). Earlier terms included “cell killing”, “antigens/peptides/epitopes”, and “gene gun”. Terms related to nonviral delivery, RNA stability, and immune-cell markers became prominent during the middle of the study period. Recent terms included “neoantigen vaccine”, “mRNA cancer vaccine”, “survival”, and “immunogenicity”. These patterns describe changes in terminology and do not by themselves indicate clinical progression.

The annual keyword heatmap showed a rapid increase in COVID-19-related terminology from 2021, followed by lower counts in 2025 and the partial 2026 period (**Figure 7C**). In contrast, “Cancer Vaccine”, “Immunotherapy”, and “Immune Checkpoint Inhibitor” increased during the later study years. “Lipid Nanoparticle” also became more frequent, whereas “Dendritic Cell” appeared throughout much of the observation period. The 20 most frequent harmonized keywords are listed in **Table 6**.

**Table 6:** Twenty most frequent harmonized keywords.

Rank	Keyword	Frequency
1	COVID-19	719
2	T Cell	389
3	mRNA Vaccine	373
4	Immunotherapy	349
5	Vaccination	315
6	COVID-19 Vaccine	265
7	Lipid Nanoparticle	204
8	RNA Vaccine	201
9	Messenger RNA	188
10	Cancer Vaccine	181
11	Immune Response	164
12	Dendritic Cell	161
13	Gamma Interferon	161
14	Tumor Necrosis Factor	149
15	Antibody	144
16	Nanoparticle	139
17	Cellular Immunity	136
18	Cancer	134
19	Immunology	134
20	Immune Checkpoint Inhibitor	120

Frequency indicates harmonized occurrences across author and database-supplied index keyword fields and does not measure topic prevalence or clinical importance.

### 3.7 BERTopic topic modeling and complementary keyword analyses

Of the 756 publications entered into the revised BERTopic analysis, 745 were assigned to five final topics and 11 remained outliers (NP = 11; 1.46% of the full corpus). Topic percentages therefore use 745 as the denominator. Topic 1 contained NP = 388 (52.1%), Topic 2 NP = 176 (23.6%), Topic 3 NP = 99 (13.3%), Topic 4 NP = 64 (8.6%), and Topic 5 NP = 18 (2.4%). Their relative positions and sizes are shown in **Figure 8A**. The 11 outliers remained in corpus-level bibliometric analyses but were excluded from topic-specific analyses (**Supplementary Table 3**).

**Figure 8:**
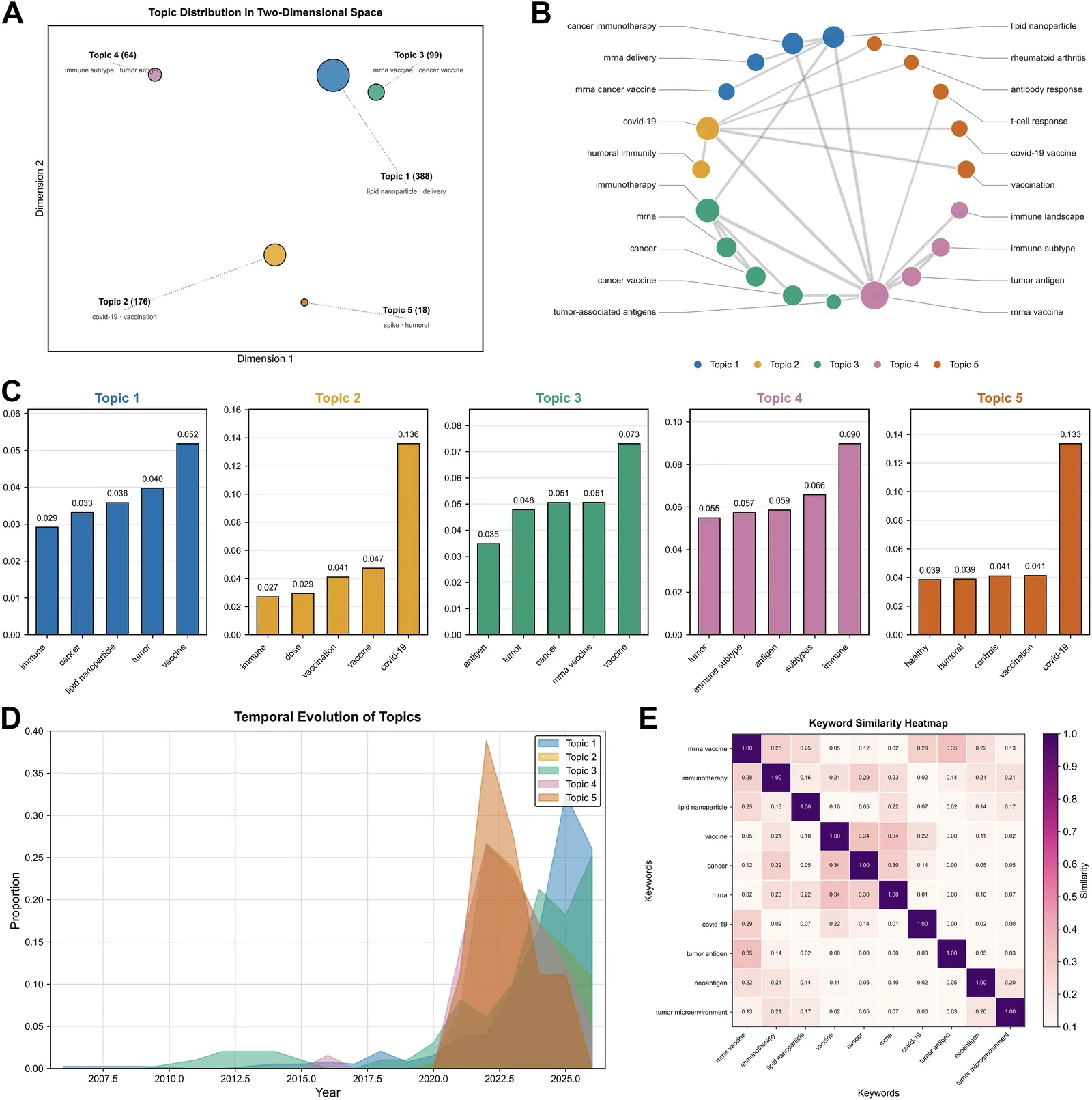
BERTopic-derived themes and complementary keyword analyses from the revised five-topic solution. **(A)** Intertopic map generated by multidimensional scaling of cosine distances between top-10 harmonized term vectors calculated with BERTopic’s standard c-TF-IDF transformation; bubble area represents topic size. **(B)** Keyword co-occurrence network based on the 745 assigned publications. The 20 nodes comprise five frequent bridge terms and three enriched terms per topic after harmonization and deduplication. Node area represents document frequency, node color identifies the topic with the highest within-topic prevalence, and edge width represents co-occurrence count. The displayed edges comprise a maximum-spanning backbone and the strongest quartile of non-zero co-occurrences. The topic-grouped circular layout is used for readability; node position does not encode semantic distance. **(C)** Five highest-weight harmonized terms per topic. **(D)** Annual publication counts normalized by each topic’s total assigned publications. **(E)** Cosine-similarity heatmap for 10 selected keyword occurrence vectors after one-to-one record linkage. Of 756 publications, 745 were assigned to five topics and 11 were primary-model outliers (1.46%).

The keyword co-occurrence network contained 20 nodes and 24 displayed edges derived from the 745 assigned publications (**Figure 8B**). “mRNA vaccine” had the highest document frequency (NP = 265). The most frequent term pairs were “mRNA vaccine”–”immunotherapy” (50 co-occurrences), “mRNA vaccine”–”COVID-19” (48), “mRNA vaccine”–”tumor antigen” (37), and “mRNA vaccine”–”lipid nanoparticle” (35). These links identify shared indexing vocabularies across vaccine platforms, oncology, immune characterization, and delivery research.

The highest-weight harmonized terms for each topic are shown in **Figure 8C**. Topic 1 emphasized vaccine, tumor, lipid nanoparticle, cancer, and immune terms and was labeled mRNA delivery and immunomodulatory cancer-vaccine research. Topic 2 emphasized COVID-19, vaccine, vaccination, dose, and immune terms and represented SARS-CoV-2 vaccination in oncology and immunotherapy settings. Topic 3 combined vaccine, mRNA vaccine, cancer, tumor, and antigen terms and represented therapeutic mRNA cancer-vaccine platforms. Topic 4 emphasized immune, subtypes, antigen, immune subtype, and tumor terms and represented tumor-antigen discovery and immune-subtype stratification. Topic 5 emphasized COVID-19, vaccination, controls, humoral, and healthy terms and represented mRNA-vaccine immunogenicity during autoimmune immunosuppression.

Within-topic annual shares are shown in **Figure 8D**. Estimates were sparse in the early years because annual NP was low. Topic 2 increased sharply during the COVID-19 period and peaked at NP = 47 in 2022. Topic 1 showed the largest absolute increase from 2024 to 2025, rising from NP = 64 to NP = 126, and remained the largest topic in the partial 2026 window. To distinguish changing topic composition from overall publication growth, annual within-year topic proportions were also calculated (**Supplementary Table 6**). Topic 2 accounted for 50.5% of assigned records in 2022, declining to 23.4% in 2024, 14.0% in 2025, and 12.8% in partial 2026. Topic 1 increased from 17.2% in 2022 to 70.4% in 2025 and 68.2% in partial 2026.

The keyword similarity heatmap (**Figure 8E**) showed the strongest selected off-diagonal similarity between “mRNA vaccine” and “tumor antigen” (0.35). “Vaccine” showed similarities of 0.34 with both “cancer” and “mRNA”, whereas “cancer” and “mRNA” had a similarity of 0.30. “COVID-19” showed modest similarity with “mRNA vaccine” (0.29) and low similarity with most other selected cancer- and delivery-related terms. This panel evaluates relationships among selected document-supplied keywords and should not be interpreted as a direct comparison of the five topic centroids.

Sensitivity analyses showed that assignment agreement varied across configurations and that the boundaries of smaller topics were parameter-dependent. The selected solution was therefore treated as an interpretable analytical summary rather than a unique taxonomy (**Supplementary Methods** and **Supplementary Table 4**). Independent two-author review of representative title–abstract records supported the five topic labels. Review of all 11 outliers indicated that they predominantly concerned infectious-disease vaccination or immune responses during immunosuppressive treatment; these records and their exclusion rationale are listed in **Supplementary Table 3**.

## 4 Discussion

### 4.1 Overview of findings

This study combined two bibliographic databases with conventional bibliometric analysis and contextual topic modeling. Publication activity increased sharply after 2020, and China and the United States accounted for the largest shares of corresponding-author publications. The geographic, institutional, and journal analyses characterized contributions across immunology, oncology, and drug-delivery research. Topic modeling further distinguished direct cancer-mRNA themes from pandemic-associated and other adjacent immune-response literature.

The temporal concurrence between rapid publication growth and the clinical deployment of COVID-19 mRNA vaccines is consistent with broader interest in mRNA platform technologies, but the present bibliometric design cannot establish causation. Similarly, differences in national or institutional output may reflect many factors, including research capacity, funding, database coverage, and collaboration patterns; these factors were not directly tested. Citation-based rankings should also be interpreted cautiously because they are affected by publication year, document type, highly cited individual papers, and the citation database used.

### 4.2 Thematic landscape and emerging research directions

#### 4.2.1 The catalytic role of COVID-19 mRNA vaccines

Topic 2 comprised NP = 176 of the 745 assigned publications (23.6%), whereas Topic 5 contained NP = 18 (2.4%). Both were related to SARS-CoV-2 mRNA vaccination, but their clinical contexts differed. Topic 2 emphasized vaccination in patients with cancer or during immunotherapy, whereas Topic 5 focused on vaccine immunogenicity during autoimmune disease and immunosuppressive treatment. In **Figure 8B**, “mRNA vaccine” co-occurred frequently with both “immunotherapy” and “COVID-19”. This pattern reflects shared platform and immune-response terminology and does not demonstrate transfer of clinical efficacy between infectious-disease and cancer settings.

The pivotal trials of BNT162b2 and mRNA-1273 established the feasibility of large-scale clinical evaluation of nucleoside-modified, lipid nanoparticle-formulated mRNA platforms (42, 43). Related advances in sequence design, manufacturing, formulation, storage, and immune monitoring are relevant to cancer-vaccine development (9–13, 44). Nevertheless, evidence from infectious-disease vaccination cannot be transferred directly to therapeutic cancer vaccination. Therapeutic vaccination must address tumor heterogeneity, tolerance, impaired antigen presentation, and local immunosuppression (23–26).

The proportion of Topic 2 declined after 2022, while cancer- and delivery-related terms remained prominent in later years. This pattern is consistent with a shift in aggregate research attention after the acute pandemic period. It does not demonstrate that the same investigators, funding streams, or technologies moved from infectious disease to oncology; confirming such transfer would require longitudinal author-, grant-, or patent-level analyses.

COVID-19, HPV, and general immunology records entered the corpus because the search required overlapping concept terms rather than a manually curated therapeutic-cancer endpoint. COVID-19 studies often included patients with cancer, T-cell responses, antigen presentation, or mRNA-platform terminology. HPV and nasopharyngeal carcinoma records linked viral antigens, tumor vaccination, and immune escape. Their prominence therefore reflects both genuine platform or immune-mechanism overlap and reduced specificity introduced by the broad immune-response block. These records should not be interpreted as direct evidence that each study evaluated an mRNA therapy for tumor immune escape.

Cellular immunity is particularly relevant to therapeutic cancer vaccines, but the underlying biology requires qualification. CD8+ T-cell recognition generally depends on peptide presentation by MHC class I molecules; loss of antigen presentation can therefore reduce, rather than facilitate, CD8+ T-cell-mediated killing (45). Strategies for tumors with impaired antigen presentation may need to restore this pathway, recruit other effector mechanisms, or exploit natural killer-cell recognition (45, 46). Programmable mRNA may support such combinations by encoding selected antigens or immunomodulatory proteins, but the effect depends on payload, delivery site, target cell, and tumor context.

#### 4.2.2 Tumor immune characterization and mRNA-based precision strategies

Topic 4 contained NP = 64 (8.6%) and was characterized by terms related to tumor subtypes, immune subtypes, tumor antigens, genes, and landscape analyses. Its position in the intertopic map was separated from the principal vaccine and immunotherapy topics. This pattern suggests that biological characterization and platform engineering formed distinguishable components of the literature, although spatial separation in a topic model does not demonstrate a lack of collaboration between the corresponding research communities.

Tumors differ in immune-cell infiltration, checkpoint expression, antigen-presentation capacity, and immunosuppressive signaling (27–31). Inflamed tumors may be more responsive to checkpoint blockade, whereas immune-desert or immune-excluded tumors often require additional approaches (5, 27–31). These differences are relevant to mRNA design. A neoantigen vaccine depends on adequate antigen processing and presentation, while an immunomodulatory payload should be selected according to the dominant resistance mechanism. Potential payloads include cytokines, costimulatory molecules, and checkpoint-blocking proteins rather than checkpoint ligands in general.

The occurrence of the term “landscape” is consistent with efforts to characterize multiple features of the tumor microenvironment rather than single markers. Such characterization may help identify combinations of antigenic and immunomodulatory payloads. However, the topic model does not show that simultaneous targeting of several pathways is always necessary or superior; that question requires experimental and clinical evaluation.

A closer connection between immune profiling and mRNA platform development may support more rational selection of payloads and delivery strategies. This interpretation remains hypothesis-generating because the current analysis did not link individual tumor profiles to treatment outcomes.

#### 4.2.3 mRNA cancer vaccines and clinical translation

Topic 3 contained NP = 99 (13.3%) and was characterized by terms related to mRNA vaccines, cancer, immune responses, and clinical evaluation. Its thematic composition supports interpretation as the most focused therapeutic mRNA cancer-vaccine component of the corpus, while its sustained representation reflects continued attention to delivery, stability, immune priming, and clinical translation.

Cancer applications pose several delivery challenges. Some approaches seek uptake by antigen-presenting cells in lymphoid tissues, whereas others aim for local or systemic delivery to tumors or immune cells in the tumor microenvironment. Extracellular nucleases, nonspecific tissue uptake, vascular and stromal barriers, and endosomal sequestration can reduce delivery efficiency (14–17). These physicochemical barriers should be distinguished from immunosuppressive mechanisms within the tumor microenvironment.

Lipid nanoparticles are the most established mRNA delivery platform, but intravenously administered formulations commonly accumulate in the liver (14–17). Modifying lipid composition, particle surface properties, and administration route may alter tissue distribution (18, 19). Passive tumor accumulation through the enhanced permeability and retention effect is variable across tumor models and patients and should not be assumed to provide reliable tumor targeting (47, 48).

Endosomal escape remains inefficient. One siRNA–LNP imaging study estimated that approximately 1–2% of internalized siRNA escaped from endosomes under the reported conditions (49). A complementary live-cell study showed that release occurred from a limited subset of endosomal compartments (50). These findings are system-specific and should not be generalized directly to mRNA–LNP formulations. A study of LNP-delivered mRNA further showed that post-endosomal trafficking and extracellular-vesicle loading can affect the fate of delivered mRNA (51). The prominence of ovalbumin (OVA) reflects its frequent use as a model antigen in preclinical studies. OVA supports mechanistic comparisons, but findings based on this model require validation with clinically relevant tumor antigens.

The trajectory of Topic 3 indicates continuing research attention to clinically oriented mRNA cancer vaccines. Progress toward clinical application will depend on balancing target-cell delivery, endosomal release, expression kinetics, immune activation, toxicity, and manufacturability (14–19, 52, 53).

#### 4.2.4 Cancer vaccines and immunotherapy as the largest thematic component

Topic 1 contained NP = 388 (52.1%) and was the largest topic in the retrieved corpus. It showed the largest absolute increase from 2024 to 2025, rising from NP = 64 to NP = 126, and remained dominant in the partial 2026 window. Its representative terms connected cancer vaccines with immunotherapy, immune responses, and delivery. Topic 1 therefore captures a broad translational theme rather than a narrowly defined biological mechanism.

The modularity of mRNA allows the expression of multiple neoantigens or immunomodulatory proteins, either within a single engineered transcript, across several transcripts, or through co-formulated products (9, 10, 15, 52–54). This flexibility may support combinations designed for heterogeneous tumors. Nanomedicine provides one means of protecting these payloads and directing them toward antigen-presenting cells or other targets, but successful delivery and immune priming depend on formulation-specific properties.

Melanoma has been a major setting for personalized neoantigen vaccination, as shown by early vaccine studies and the KEYNOTE-942 trial (23–26, 53–55). In the randomized phase 2b KEYNOTE-942 study, the personalized mRNA vaccine mRNA-4157 (V940), which encodes up to 34 neoantigens, was evaluated with pembrolizumab after resection of high-risk melanoma; the reported relative reduction in the risk of recurrence or death was approximately 44% (55). This result should not be generalized to other tumors. Personalized RNA vaccination has also been evaluated in pancreatic ductal adenocarcinoma, where a phase 1 study reported vaccine-induced T-cell responses in a subset of patients (56).

The keyword “neoadjuvant” indicates interest in administering immunotherapy before surgical resection. Because the present analysis did not distinguish mRNA vaccination from other neoadjuvant approaches, it cannot establish whether neoadjuvant mRNA vaccination improves clinical outcomes or reduces immune escape.

Unlike the pandemic-associated topic, Topic 1 increased during the most recent complete years. This descriptive trajectory is compatible with growing clinical and translational interest, but it does not establish clinical maturity. Outcomes from ongoing trials, manufacturing time, cost, quality control, and regulatory requirements will influence broader implementation of personalized mRNA vaccines (52, 53).

### 4.3 Implications and future directions

The bibliometric and topic-modeling analyses provide value for their intended purpose. They organize a heterogeneous literature, distinguish directly relevant cancer-mRNA research from adjacent platform and immune-response studies, and identify concentrations and gaps across delivery, antigen selection, immune context, collaboration, and citation structure. This evidence map can guide targeted systematic reviews, experimental comparisons, trial-registry analyses, and research partnerships. Its purpose is to prioritize and structure subsequent evidence generation, not to substitute for mechanistic experiments or clinical trials.

First, the recent growth of the cancer-oriented Topics 1 and 3 supports continued investigation of the interface between neoantigen selection and delivery-system design. Publication growth identifies an active research area, but research priority should also be guided by biological rationale, unmet need, and clinical evidence.

Second, the separation of immune-characterization and platform topics suggests a potential opportunity to connect tumor profiling with payload and formulation selection. Because this inference is based on topic structure rather than direct project-level analysis, it should be tested using experimental, clinical, or collaboration data.

Third, the temporal pattern is compatible with, but does not establish, knowledge transfer from infectious-disease mRNA platforms to oncology. Recent synthesis of mRNA vaccine development describes advances in sequence engineering, delivery, manufacturing, and clinical implementation that may inform cancer-vaccine research (57). This interpretation remains hypothesis-generating; author-, patent-, grant-, manufacturing-, or trial-level analyses would be required to demonstrate transfer.

Fourth, delivery remains a persistent research focus. Relevant challenges include limiting unwanted hepatic accumulation, improving cytoplasmic release, identifying appropriate target cells, and co-delivering antigenic and immunomodulatory payloads without excessive toxicity.

Clinical translation also requires timely tumor sequencing, reproducible neoantigen selection, rapid manufacturing, and validated quality-control procedures (52, 53). Tumor immune escape mechanisms differ among patients and cancer types, making a universally effective construct unlikely. Trials may therefore benefit from incorporating immune and molecular profiling while avoiding *post hoc* subgroup claims. Regulatory pathways for individualized products also require continued development.

The biological interpretation also depends on three coupled processes. First, delivery kinetics determine which cells translate the payload, the duration of antigen expression, and the balance between immune priming and off-target inflammation (14–19). Second, neoantigen-selection algorithms must integrate tumor clonality, transcript expression, HLA presentation, and immunogenicity, and predicted candidates still require experimental validation (52–54). Third, vaccine-expanded T cells may remain constrained by PD-1/PD-L1 or CTLA-4 signaling, impaired MHC class I presentation, regulatory T cells, suppressive myeloid cells, and other features of the tumor microenvironment (1–5, 27–31). The thematic convergence of delivery, neoantigen selection, and immune characterization therefore motivates combination strategies, but the bibliometric analysis cannot determine which combination is effective.

Single-cell and spatial profiling can improve biological characterization of the tumor microenvironment, whereas topic modeling can map the published literature; these methods address different levels of evidence and should not be treated as interchangeable. Systematic reviews and trial-registry analyses, rather than bibliometric trends alone, are needed to assess clinical benefit. Future platform research may examine tissue-selective delivery, stimulus-responsive release, neoantigen prediction, and sequence optimization, with performance evaluated in clinically relevant models.

### 4.4 Limitations

This study has several limitations. First, the search was restricted to WoSCC and Scopus and to English-language Articles and Reviews. Relevant publications indexed elsewhere or published in other languages may therefore have been missed. The fixed 2006 start date also excludes earlier foundational mRNA literature. Second, the broad Boolean concepts captured both directly relevant cancer-mRNA research and adjacent platform or immune-response literature. The study is therefore framed as an exploratory map of the retrieved cross-domain corpus. Third, the 2026 data were incomplete, and citation indicators were affected by publication age, document type, database coverage, and highly cited papers. The citations-per-year check reduced but did not eliminate these differences, and citation counts retained from WoSCC and Scopus are not strictly interchangeable. Fourth, country participation, institutional full counting, and incomplete author disambiguation may inflate contributions or combine different individuals. Fifth, BERTopic results depend on preprocessing, embeddings, dimensionality reduction, clustering, and manual labeling. Sensitivity analyses indicated that smaller-topic boundaries were configuration-dependent, so the selected solution is interpreted as one analytical partition rather than a unique taxonomy. **Figure 8B** displays a filtered co-occurrence backbone for readability, so an undisplayed edge does not indicate absence of co-occurrence. Finally, bibliometric activity and citation impact do not measure methodological rigor, therapeutic efficacy, or clinical benefit.

## 5 Conclusions

Publication activity in the retrieved cross-domain corpus increased substantially after 2020. The revised five-topic analysis mapped mRNA delivery and immunomodulatory cancer vaccines, SARS-CoV-2 vaccination in oncology, therapeutic mRNA cancer-vaccine platforms, tumor-antigen and immune-subtype studies, and vaccine immunogenicity during autoimmune immunosuppression. By locating connections among delivery, antigen selection, immune context, and geographic collaboration, this map provides a rational starting point for focused systematic reviews, experimental studies, and clinical evaluation. Bibliometric evidence prioritizes questions and reveals knowledge structure; it does not by itself establish that a particular mRNA strategy reverses tumor immune escape or improves clinical outcomes.

## Data Availability

The datasets presented in this article are not readily available because the bibliographic records analyzed in this study were obtained from the Web of Science Core Collection and Scopus under database-specific institutional access agreements and cannot be publicly redistributed by the authors. Derived results supporting the findings are included in the article and **Supplementary Information**. The analysis workflow is described in the Methods and **Supplementary Methods**, including software versions, preprocessing rules, model parameters, and sensitivity configurations. BERTopic is an established open-source package with publicly available documentation. Study-specific scripts and shareable derived files are available from the corresponding authors for reasonable academic purposes, subject to applicable database licences, data-use conditions, and confidentiality requirements. No public DOI-bearing repository is claimed for the study-specific workflow. Requests to access the datasets should be directed to Xiao-Jie Zhou, zhouxj_edu@outlook.com.
