# Supplementary material for: Mapping programmable mRNA research relevant to cancer-associated immune escape: a dual-database bibliometric and topic modeling analysis
Source: Front Oncol. 2026 Oct 7;16:1957469. doi: 10.3389/fonc.2026.1957469 (PMC13683939; doi:10.3389/fonc.2026.1957469)
Supplement: Supplementary file 1 [file Table1.docx]

**Supplementary Information**

**Mapping Programmable mRNA Research Relevant to Cancer-Associated Immune Escape: A Dual-Database Bibliometric and Topic Modeling Analysis**

**Supplementary Table 1. Database-specific search strategies**

Both searches were conducted on July 29, 2026, and covered publications from January 1, 2006, through the search date.

| **Database component** | **Specification** |
| --- | --- |
| WoSCC: field and scope | Topic Search (TS), which searches titles, abstracts, author keywords, and Keywords Plus. Eligible records were English-language Articles and Reviews. |
| WoSCC: complete query | TS=("programmable mRNA" OR "engineered mRNA" OR "modified mRNA" OR "mRNA therapeutics" OR "mRNA-based therapy" OR "mRNA vaccine*" OR "mRNA immunotherapy" OR "mRNA cancer vaccine*" OR "mRNA tumor vaccine*" OR "neoantigen mRNA") AND (cancer* OR tumor* OR tumour* OR carcinoma* OR melanoma* OR neoplasm*) AND ("immune escape" OR "immune evasion" OR "tumor immune escape" OR "immune checkpoint*" OR "checkpoint blockade" OR "PD-1" OR "PD-L1" OR "CTLA-4" OR "T cell activation" OR "T cell response" OR "antigen presentation" OR "MHC class I" OR "tumor immunogenicity") |
| Scopus: field and scope | TITLE-ABS-KEY, which searches titles, abstracts, and indexed keyword fields. Eligible records were English-language Articles and Reviews. |
| Scopus: complete query | TITLE-ABS-KEY(("programmable mRNA" OR "engineered mRNA" OR "modified mRNA" OR "mRNA therapeutics" OR "mRNA-based therapy" OR "mRNA vaccine*" OR "mRNA immunotherapy" OR "mRNA cancer vaccine*" OR "mRNA tumor vaccine*" OR "neoantigen mRNA") AND (cancer* OR tumor* OR tumour* OR carcinoma* OR melanoma* OR neoplasm*) AND ("immune escape" OR "immune evasion" OR "tumor immune escape" OR "immune checkpoint*" OR "checkpoint blockade" OR "PD-1" OR "PD-L1" OR "CTLA-4" OR "T cell activation" OR "T cell response" OR "antigen presentation" OR "MHC class I" OR "tumor immunogenicity")) |

**Supplementary Table 2. Completed BIBLIO checklist for reporting bibliometric reviews of the biomedical literature**

Rights and permissions: The original checklist was published by Montazeri A, Mohammadi S, Hesari PM, Ghaemi M, Riazi H, and Sheikhi-Mobarakeh Z. Preliminary guideline for reporting bibliometric reviews of the biomedical literature (BIBLIO): a minimum requirements. Systematic Reviews. 2023;12:239. doi: 10.1186/s13643-023-02410-2. The article is licensed under the Creative Commons Attribution 4.0 International License (http://creativecommons.org/licenses/by/4.0/). Changes were limited to adding the full references cited in the checklist and completing the reporting-location column.

| **Section/Topic** | **Item No.** | **Checklist item** | **Reported on page No.** |
| --- | --- | --- | --- |
| Title |  |  |  |
| Identification | 1 | Identify the report as a bibliometric review in the title. | Main manuscript p. 1 (Title) |
| Issues/topics | 2 | Indicate the key issues/topics under investigation and coverage of time period. | Main manuscript pp. 1–2 and 7–8 (Title, Abstract, Methods 2.1) |
| Abstract |  |  |  |
| Structured summary | 3 | Structured summary including (as applicable): background, methods, results (key findings) and conclusions. | Main manuscript pp. 1–2 (Abstract) |
| Introduction/ Background |  |  |  |
| Justification/ Rationale/ Explanation | 4 | Present review of existing knowledge and epidemiological information. | Main manuscript pp. 5–7 (Introduction) |
| Objectives | 5 | Statement of the objective (s) or question (s). | Main manuscript p. 7 (end of Introduction) |
| Methods |  |  |  |
| Search engines (data sources) | 6 | Describe all information sources (such as electronic databases, contact with study authors, trial registers or other grey literature sources). | Main manuscript pp. 7–8 (Methods 2.1) |
| Search strategy | 7 | Keywords and systematization criteria (date of search, language, type of document) for the search. | Main manuscript pp. 7–8 (Methods 2.1); Supplementary Table 1 |
| Time period | 8 | The period that the review covers and the justification. | Main manuscript pp. 7–8 and 26 (Methods 2.1; Limitations) |
| Eligibility criteria | 9 | Describe all inclusion and exclusion criteria; languages; study design, type of publication and time period. | Main manuscript pp. 7–8 (Methods 2.1) |
| Data refinement (data selection procedure) | 10 | Remove the irrelevant articles; inspection to eliminate duplicate and unrelated articles (after evaluation of the title, abstract and content). | Main manuscript pp. 7–8 and 35 (Methods 2.1; Figure 1 legend) |
| Quality assessment (optional) | 11 | Assessment of papers by three authors and the use of assessing checklists. | Not applicable as a study-quality appraisal; qualitative topic checks are reported on main manuscript p. 12 and in the Supplementary Methods and Supplementary Table 3 |
| Data synthesis | 12 | Describe the methods used for summarizing, handling, synthesis, tabulations or schematic displays. Describe how the data were analysed. | Main manuscript pp. 8–12 (Methods 2.2–2.4); Supplementary Methods |
| Results |  |  |  |
| Descriptive findings (statistics) | 13 | - Provide details of the search and selection process in a flow diagram. - Number of citations retrieved (number of publication, year of publication, type of documents, country of publication, articles with the highest impact, most impactful authors, most impactful articles, authors with the highest production, top journals, top institutions, …) | Main manuscript pp. 13–19 (Results); Figure 1 legend and Tables 1–6 |
| Schematic map and trend | 14 | Summarize and/or present the schematic maps and trends using an appropriate software to present citations, journals, authors, top journals, time trends, emerging literature, and any relevant indicators (as applicable) [1-5]. | Main manuscript pp. 8–19 and 35–37 (Methods 2.2–2.4; Results; Figures 2–8) |
| Tabulation and summarizing the findings | 15 | General recommendation: Studies under consideration could be summarized and organized by different subtitles and different scenarios. Regardless, results need to be presented in separate tables covering each subtitle. The followings are some options that could help to summarize the findings.  Option 1: - Start the presentation with a historical view [when and who first published on the topic]. - Report on review papers. The result should be listed in a separate table. Also, specify the review type (scoping review, narrative review, systematic review, and meta-analysis). - Summarize the findings according to the study designs and main study types. Option 2: - Start the presentation with a historical view [when and who first published on the topic]. - Report on review papers. The result should be listed in a separate table. Also, indicate the review type (scoping review, narrative review, systematic review, and meta-analysis) should be specified.  - Summarize the findings according to outcome measures or populations. For example, see [6]. Option 3: - Start the presentation with a historical view [when and who first published on the topic]. - Report on review papers. The result should be listed in a separate table. Also, specify the review type (scoping review, narrative review, systematic review, and meta-analysis). - Summarize the findings according to concept [7]. Option 4. - Start the presentation with a historical view [when and who first published on the topic]. - Report on review papers. The result should be listed in a separate table, and also specify the review type (scoping review, narrative review, systematic review, and meta-analysis). - Summarize the findings according to different subtitles relevant to the main topic [8]. | Main manuscript pp. 13–19 and 38–44 (Results; Tables 1–6); Supplementary Tables 1–6 |
| Synthesis of findings | 16 | Synthesize the findings as much as possible, find the gap, and propose a model, hypothesis, etc. (if applicable). | Main manuscript pp. 19–26 (Discussion 4.1–4.3) |
| Discussion |  |  |  |
| Summary of evidence | 17 | Summarize the main findings. The findings should be presented in more "general" or "accessible" terms. | Main manuscript p. 19 (Discussion 4.1) |
| Interpretation | 18 | Include interpretation consistent with results. Explanations for observed outcomes, similarities, and differences reported would be essential. | Main manuscript pp. 19–26 (Discussion 4.2–4.3) |
| Strengths and limitations | 19 | Discuss the strengths and limitations. | Main manuscript p. 26 (Limitations) |
| Conclusion(s) | 20 | Provide a general interpretation of the results with respect to the review questions and objectives, as well as potential implications. | Main manuscript p. 27 (Conclusions) |

**References cited in the BIBLIO checklist**
1. McDougal L, Dehingia N, Cheung WW, Dixit A, Raj A. COVID-19 burden, author affiliation and women's well-being: A bibliometric analysis of COVID-19 related publications including focus on low-and middle-income countries. eClinicalMedicine 2022; 52: 101606.
2. Henstock L, Wong R, Tsuchiya A, Spencer A. Behavioral theories that have influenced the way health state preferences are elicited and interpreted: A bibliometric mapping analysis of the ttime trade-off method with VOSviewer visualization. Front Health Serv 2022; 2: 848087.
3. Bodea F, Bungau SG, Negru AP, Radu A, Tarce AG, Tit DM, et al. Exploring new therapeutic avenues for ophthalmic disorders: Glaucoma-related molecular docking evaluation and bibliometric analysis for improved management of ocular diseases. Bioengineering 2023; 10(8): 983.
4. Sang XZ, Wang CQ, Chen W, Rong H, Hou LJ. An exhaustive analysis of post-traumatic brain injury dementia using bibliometric methodologies. Front Neurol 2023; 14: 1165059.
5. Ramli MI, Hamzaid NA, Engkasan JP, Usman J. Respiratory muscle training: a bibliometric analysis of 60 years’ multidisciplinary journey. Biomed Eng Online 2023; 22(1): 50.
6. Akosman I, Kumar N, Mortenson R, Lans A, De La Garza Ramos R, Eleswarapu A,et al. Racial differences in perioperative complications, readmissions, and mortalities after elective spine surgery in the United States: A systematic review using AI-assisted bibliometric analysis. Glob Spine J 2023: 21925682231186759.
7. Tavousi M, Mohammadi S, Sadighi J, Zarei F, Kermani RM, Rostami R, Montazeri A. Measuring health literacy: A systematic review and bibliometric analysis of instruments from 1993 to 2021. Plos One 2022; 17(7): e0271524.
8. Montazeri A. Health-related quality of life in breast cancer patients: A bibliographic review of the literature from 1974 to 2007. J Exp Clin Cancer Res 2008; 27: 32.

**Supplementary Methods. BERTopic model specification, qualitative review, and robustness analysis**

The reference BERTopic analysis used all-MiniLM-L6-v2 document embeddings. UMAP reduced the embeddings using 15 nearest neighbors, five output dimensions, zero minimum distance, cosine distance, and initialization seed 42.

HDBSCAN used a minimum cluster size of 12, a minimum of five samples, Euclidean distance, excess-of-mass cluster selection, and prediction support. Topic descriptors included one- and two-word terms occurring in at least two documents and no more than 90% of documents. The topic-term matrix was transformed with BERTopic’s ClassTfidfTransformer: term counts were normalized within each topic, and inverse-frequency weights used the term’s total frequency across all topic-aggregated documents. For Figure 8A and Figure 8C, documented lexical variants were first merged at the count-matrix stage and the same c-TF-IDF transformation was then applied.

The dominant 487-record cluster was reanalyzed using 10 nearest neighbors, three output dimensions, and a minimum cluster size of 60. The remaining dimensionality-reduction and clustering choices were unchanged. The secondary model initially labelled 62 records as low-density outliers. Because these records belonged to the coherent dominant primary cluster, they were transformed against the fitted secondary model. Records still labelled as outliers were assigned to the secondary topic with the highest membership probability. This post-clustering assignment was tracked separately. The 11 primary-model outliers were not reassigned. The two-stage procedure therefore assigned 745 records to five topics and retained 11 primary-model outliers.

For document-supplied keyword analyses, source keyword fields were linked to topic assignments with a stable record key. A normalized DOI was used when available; otherwise, a SHA-256 hash of the normalized title, publication year, and abstract was used. Keys were required to be unique in both files, and the merge was validated as one-to-one. This rule preserved all four records without a DOI as distinct publications.

Robustness was assessed across five prespecified configurations that varied the initialization seed and minimum cluster size. The originally submitted blanket token-length filter produced three non-outlier topics and no outliers. Its agreement with the entity-aware solution was moderate (ARI, 0.581; NMI, 0.659).

**Qualitative review of topic labels and outliers**

Two authors independently examined representative titles and abstracts from each of the five final topics, compared them with the corresponding weighted terms and provisional labels, and resolved differences by discussion. Consensus review supported the five labels and the distinction between cancer-focused topics and adjacent vaccine or immunosuppression literature.

The same two authors independently reviewed all 11 primary-model HDBSCAN outliers and resolved differences by discussion. These records were retained in corpus-level bibliometric analyses but excluded from topic-specific analyses because BERTopic did not assign them to a sufficiently dense cluster. Their content mainly concerned infectious-disease vaccination or vaccine immune responses during immunosuppressive treatment, as detailed in Supplementary Table 3.

**Supplementary Table 3. BERTopic outliers excluded from topic-specific analyses**

| **Record** | **Article title** | **Reason for exclusion from topic-specific analyses** |
| --- | --- | --- |
| 1. 2025 10.3389/fimmu.2025.1682049 | Minimal association between Th1-specific responses to COVID-19 vaccines and SARS-CoV-2 breakthrough infections in multiple sclerosis patients receiving disease-modifying therapies | COVID-19 vaccine T-cell responses in MS during DMT; adjacent immunosuppression literature. |
| 2. 2023 10.1136/jnnp-2022-330175 | Longitudinal characterisation of B and T-cell immune responses after the booster dose of COVID-19 mRNA-vaccine in people with multiple sclerosis using different disease-modifying therapies | COVID-19 mRNA-vaccine responses in MS during DMT; adjacent immunosuppression literature. |
| 3. 2024 10.3390/vaccines12060684 | SARS-CoV-2-Specific Immune Cytokine Profiles to mRNA, Viral Vector and Protein-Based Vaccines in Patients with Multiple Sclerosis: Beyond Interferon Gamma | SARS-CoV-2 vaccine cytokine responses in MS; adjacent immunosuppression literature. |
| 4. 2025 10.1002/eji.202451142 | Distinct Requirements for CD4+ T Cell Help for Immune Responses Induced by mRNA and Adenovirus-Vector SARS-CoV-2 Vaccines | CD4+ T-cell help after SARS-CoV-2 vaccination; infectious-disease vaccine immunology without a cancer focus. |
| 5. 2025 10.1016/j.jneuroim.2025.578694 | SARS-CoV-2 spike antibody and T cell response in MS patients on high efficacy therapies post vaccine | SARS-CoV-2 vaccine responses in MS receiving high-efficacy therapy; adjacent immunosuppression literature. |
| 6. 2024 10.1080/22221751.2023.2292068 | Concomitant administration of seasonal influenza and COVID-19 mRNA vaccines | Concomitant influenza and COVID-19 vaccination; infectious-disease vaccination without a cancer focus. |
| 7. 2022 10.1002/ana.26343 | Severe Acute Respiratory Syndrome Coronavirus 2 Third Vaccine Immune Response in Multiple Sclerosis Patients Treated with Ocrelizumab | Third-dose SARS-CoV-2 vaccine response in ocrelizumab-treated MS; adjacent immunosuppression literature. |
| 8. 2022 10.3389/fimmu.2022.1050183 | Multiple sclerosis-disease modifying therapies affect humoral and T-cell response to mRNA COVID-19 vaccine | COVID-19 mRNA-vaccine responses during MS therapy; adjacent immunosuppression literature. |
| 9. 2021 10.1038/s41591-021-01507-2 | Cellular and humoral immune responses following SARS-CoV-2 mRNA vaccination in patients with multiple sclerosis on anti-CD20 therapy | SARS-CoV-2 mRNA-vaccine responses during anti-CD20 treatment for MS; adjacent immunosuppression literature. |
| 10. 2019 10.1016/j.omtm.2018.10.010 | Efficient Induction of T Cells against Conserved HIV-1 Regions by Mosaic Vaccines Delivered as Self-Amplifying mRNA | Self-amplifying mRNA vaccination against HIV-1; infectious-disease vaccine research without a cancer focus. |
| 11. 2023 10.1128/spectrum.04240-22 | mRNA Vaccine Mitigates SARS-CoV-2 Infections and COVID-19 | Preclinical SARS-CoV-2 mRNA-vaccine development; infectious-disease vaccine research without a cancer focus. |

Note: These 11 records remained part of the 756-record corpus for non-topic bibliometric analyses. Their outlier status reflects density-based topic assignment rather than removal during database screening. MS, multiple sclerosis; DMT, disease-modifying therapy.

**Supplementary Table 4. Sensitivity of BERTopic topic assignments to model initialization and minimum cluster size**

| **Configuration** | **Seed** | **Minimum cluster size** | **Topics** | **Outliers** | **ARI vs common comparison** | **NMI vs common comparison** |
| --- | --- | --- | --- | --- | --- | --- |
| Selected solution | 42 | 12 | 5 | 11 | 0.959 | 0.945 |
| Common comparison | 42 | 15 | 4 | 0 | 1.000 | 1.000 |
| Cluster-size sensitivity | 42 | 18 | 4 | 0 | 1.000 | 1.000 |
| Seed sensitivity | 21 | 15 | 6 | 3 | 0.521 | 0.700 |
| Seed sensitivity | 84 | 15 | 6 | 0 | 0.602 | 0.756 |

Note: ARI, adjusted Rand index; NMI, normalized mutual information. Values compare each configuration with the common seed-42, minimum-cluster-size-15 solution. The selected solution was retained because it provided the five-topic partition used for interpretation. Identical values for the common and cluster-size-18 configurations indicate unchanged assignments despite the parameter change.

**Supplementary Table 5. Age-standardized citation ranking of the leading journals**

| **Rank** | **Journal** | **Summed citations per year** |
| --- | --- | --- |
| 1 | Nature | 505.75 |
| 2 | Molecular Therapy | 298.98 |
| 3 | Frontiers in Immunology | 251.10 |
| 4 | Signal Transduction and Targeted Therapy | 202.42 |
| 5 | Proceedings of the National Academy of Sciences of the United States of America | 200.66 |
| 6 | Molecular Cancer | 192.58 |

Note: For each record, citations per year were calculated as total citations divided by (2026 minus publication year plus 1). Values were then summed by standardized journal title. Citation counts were retained from the source record after cross-database deduplication and are not a strictly single-database measure.

**Supplementary Table 6. Annual counts and within-year proportions for the five BERTopic topics**

Annual topic counts and within-year proportions are reported below. Each proportion uses all assigned publications in the corresponding calendar year as the denominator and excludes the 11 outliers. The 2026 row covers the partial year through July 29, 2026.

| **year** | **Assigned records** | **Topic 1 count** | **Topic 1 proportion (%)** | **Topic 2 count** | **Topic 2 proportion (%)** | **Topic 3 count** | **Topic 3 proportion (%)** | **Topic 4 count** | **Topic 4 proportion (%)** | **Topic 5 count** | **Topic 5 proportion (%)** |
| --- | --- | --- | --- | --- | --- | --- | --- | --- | --- | --- | --- |
| 2006 | 1 | 1 | 100 | 0 | 0 | 0 | 0 | 0 | 0 | 0 | 0 |
| 2009 | 1 | 1 | 100 | 0 | 0 | 0 | 0 | 0 | 0 | 0 | 0 |
| 2011 | 1 | 0 | 0 | 0 | 0 | 1 | 100 | 0 | 0 | 0 | 0 |
| 2012 | 2 | 0 | 0 | 0 | 0 | 2 | 100 | 0 | 0 | 0 | 0 |
| 2014 | 4 | 2 | 50 | 0 | 0 | 2 | 50 | 0 | 0 | 0 | 0 |
| 2015 | 3 | 2 | 66.7 | 0 | 0 | 1 | 33.3 | 0 | 0 | 0 | 0 |
| 2016 | 4 | 3 | 75 | 0 | 0 | 0 | 0 | 1 | 25 | 0 | 0 |
| 2017 | 2 | 2 | 100 | 0 | 0 | 0 | 0 | 0 | 0 | 0 | 0 |
| 2018 | 9 | 8 | 88.9 | 0 | 0 | 1 | 11.1 | 0 | 0 | 0 | 0 |
| 2019 | 4 | 3 | 75 | 0 | 0 | 1 | 25 | 0 | 0 | 0 | 0 |
| 2020 | 10 | 6 | 60 | 1 | 10 | 3 | 30 | 0 | 0 | 0 | 0 |
| 2021 | 46 | 15 | 32.6 | 12 | 26.1 | 8 | 17.4 | 9 | 19.6 | 2 | 4.3 |
| 2022 | 93 | 16 | 17.2 | 47 | 50.5 | 6 | 6.5 | 17 | 18.3 | 7 | 7.5 |
| 2023 | 110 | 38 | 34.5 | 42 | 38.2 | 10 | 9.1 | 15 | 13.6 | 5 | 4.5 |
| 2024 | 128 | 64 | 50 | 30 | 23.4 | 21 | 16.4 | 11 | 8.6 | 2 | 1.6 |
| 2025 | 179 | 126 | 70.4 | 25 | 14 | 18 | 10.1 | 8 | 4.5 | 2 | 1.1 |
| 2026 | 148 | 101 | 68.2 | 19 | 12.8 | 25 | 16.9 | 3 | 2 | 0 | 0 |

**Notes**

Item Definition

Denominator All BERTopic-assigned publications in the same calendar year.

Outliers The 11 BERTopic outliers are excluded from topic-specific annual proportions.

Interpretation Each annual row sums to 100% across Topics 1–5, subject to rounding.

Partial year The 2026 values cover January 1 through July 29, 2026.
